# Personal Glucose Meter: Biosensing Platforms for Environmental Toxicants

**DOI:** 10.3390/bios15120811

**Published:** 2025-12-13

**Authors:** Elena Dorozhko, Anna Solomonenko, Alena Koltsova, Elena Korotkova, Ekaterina Mikhnevich, Mrinal Vashisth, Pradip Kar, Amrit Hui, Muhammad Saqib

**Affiliations:** 1 Chemical Engineering Division, School of Earth Sciences and Engineering, National Research Tomsk Polytechnic University, 30 Lenin Avenue, 634050 Tomsk, Russia; 2 National Research Tomsk State University, 36 Lenin Avenue, 634050 Tomsk, Russia; 3 Parasitology Laboratory, Department of Zoology, Cooch Behar Panchanan Barma University, Vivekananda Street, Cooch Behar 736101, India; 4 UNESCO Laboratory of Environmental Electrochemistry, Department of Analytical Chemistry, Faculty of Science, Charles University, Hlavova 8/2030, CZ 128 43 Prague, Czech Republic

**Keywords:** personal glucose meter, biosensor, point-of-care diagnostics, environmental monitoring, ecotoxicants

## Abstract

The detection of environmental toxicants is transitioning from centralized laboratory methods to decentralized, point-of-care (POC) monitoring. A highly innovative approach in this field is the repurposing of commercially available, low-cost, and portable personal glucose meters (PGMs) as universal biosensing platforms. This strategy leverages the widespread availability and ease of use of PGMs to develop rapid, on-site detection methods for a wide array of non-glucose targets, significantly reducing both cost and development time. This systematic review comprehensively examines the various strategies employed to adapt PGMs for the detection of a wide array of ecotoxicants, including chemical targets (antibiotics, mycotoxins, pesticides, heavy metals, persistent organic pollutants) and biological ones (pathogenic bacteria, and viruses). The systematic review critically evaluates different sensor designs, highlighting that while aptamer-based and non-enzymatic biosensors offer advantages in stability and cost, antibody-based sensors provide high specificity. A significant finding is the persistent trade-off between analytical sensitivity and practical field deployment; many of the most sensitive assays require multi-step procedures, precise temperature control, magnetic separation, centrifugation, and the use of additional equipment, factors that undermine true POC utility. To address this gap, we propose four essential criteria for POC readiness: (i) ambient-temperature operation, (ii) no reliance on magnetic or centrifugal separation, (iii) total assay time, and (iv) robustness in complex environmental matrices. This systematic review confirms the feasibility of this approach across a broad spectrum of targets. However, the key challenge for future research lies in simplifying the assay protocols, eliminating cumbersome sample preparation steps, and enhancing robustness to make these biosensors truly practical for routine, on-site environmental monitoring.

## 1. Introduction

Despite the predominance of the centralized laboratory detection of ecotoxicants, biosensor methods are approaching the era of decentralized monitoring. The rapid, reliable and cost-effective detection of biological and synthetic pollutants is now possible in places other than laboratories, such as mobile laboratories or at home in a point-of-care (POC) setting [1]. Miniature and portable devices are particularly useful for POC mode, as they can easily be used by individuals without the specialized education required to operate complex and bulky devices used in centralized laboratories. Adapting off-the-shelf devices to detect various pollutants is one of the most innovative approaches in developing new POC testing technologies, significantly reducing both cost and development time.

To date, the repurposing of personal glucose meters (PGM) has become particularly popular for converting commercial portable devices to analyze various substances [1,2,3]. The primary purpose of PGM is the routine monitoring of blood glucose levels at home or in a doctor’s office to detect diabetes mellitus [4]. However, adapting commercial PGMs for environmental monitoring requires the careful consideration of their design and operating principle.

Most modern glucose meters are based on an electrochemical method in which enzymes (glucose oxidase (GOx)) accelerate the chemical reaction between glucose and a mediator molecule, usually ferricyanide or a complex of osmium bipyridyl [5]. The mediator transfers electrons to the electrode, generating an electric current [6,7]. The glucose meter displays the glucose level in mg·L^−1^ or mmol·L^−1^ units within a few seconds [8]. Glucose is oxidized to gluconic acid or gluconolactone and hydrogen peroxide in GOx-based glucose meters [5,9]. The hydrogen peroxide formed is then determined amperometrically [5]. GOx-based glucose meters are more sensitive to the concentration of oxygen, ascorbic and uric acids, and acetaminophen in the analyzed sample [9]. GDH catalyzes the oxidation of glucose using cofactors such as NAD^+^, pyrroloquinoline-quinoline-quinoline (PQQ) or FAD, and these systems are less oxygen-dependent [10]. Some devices using the GDH-PQQ system for glucose determination may give falsely overestimated results due to reactions to other sugars such as maltose and galactose, which are found in some foods and medicines [5]. The authors of the publication [11] note that before using PGM to analyze a sample it must be calibrated in the presence of the sample matrix components to reduce the measurement error of the analytical signal. The authors also carefully review the existing commercial PGMs on the market with accuracy indicators [11].

Despite their widespread availability and user-friendly operation, repurposed PGMs face several inherent limitations that complicate their adaptation for non-glucose analytes. First, many environmental and food samples contain endogenous glucose or other reducing substances (e.g., ascorbic acid, uric acid), which can generate high background signals and compromise assay specificity [9,11]. Second, commercial test strips are engineered for a narrow physiological glucose range and operate within a fixed electrochemical window optimized for blood matrices, making them poorly suited for detecting subtle signal changes in complex environmental samples [5,11]. Third, enzymatic labels (e.g., invertase, GOx) commonly used to transduce target binding into glucose signals are prone to denaturation under field conditions, particularly in the presence of pH shifts, temperature fluctuations, or inhibitory compounds such as heavy metals or phenolics [9]. These constraints necessitate careful assay design often involving sample pretreatment, signal amplification, or separation steps which can undermine the very simplicity that makes PGMs attractive for point-of-care use. Recognizing these challenges is essential to critically evaluate the true practicality of reported biosensing strategies.

The simplest strategy was to use enzymes to convert glucogenic substrates into glucose, which allowed the reuse of PGM. For such purposes, invertase is often used; less often used are alkaline phosphatase (ALP) and glucoamylase. In order to ensure the selectivity of the detection of both synthetic and biological ecotoxicants in different analyzed objects, conjugates based on enzymes and magnetic nanoparticles (for example, Fe_2_O_3_ NPs) are preliminarily created with specific receptors grafted on antibodies, aptamers obtained using SELEX technology, DNA, and cells. Magnetic nanoparticles open up wide application prospects due to their unique magnetic separation ability, which makes it possible to effectively reduce the influence of interfering components in the studied samples. Some works are devoted to research on the use of nanozymes, nanomaterials with enzyme-like activity, or DNAzymes, short single-stranded DNA molecules with catalytic activity, in biosensors instead of enzymes. As a rule, the operation of biosensors based on nanosimes or DNAzymes is based on the oxidation of glucose as a substrate and a decrease in the analytical signal of PGM.

A more advanced strategy bypasses the invertase-mediated reaction by using NAD-dependent enzymes to catalyze the formation of NADH, which directly triggers a signal in the, or biosensors based on compounds that directly affect the mediator system of the PGM. This discovery further expands the versatility of PGM in detecting a wider range of ecotoxicants. The article also describes an innovative approach due to its combination with nucleic acid and Cas12a amplification systems for the detection of both biological ecotoxicants (bacteria, viruses) and inorganic toxicants (heavy metals). However, the implementation of these approaches usually requires cumbersome conjugation of DNA with enzymes and complex sample processing, including additional equipment for thermostating and centrifugation during separation of the target analyte. An approach involving the targeted release of glucose-related enzymes from nanocontainers such as hydrogels and liposomes is also noted. While several previous reviews have summarized the repurposing of personal glucose meters (PGMs) for biomedical diagnostics. Moreover, existing reviews tend to focus on technical feasibility, often overlooking critical practical barriers to field deployment.

This systematic review uniquely bridges this gap. Our work is the first to (i) provide a comprehensive, evidence based mapping of PGM-based sensors and biosensors for a wide spectrum of environmental toxicants, spanning antibiotics, mycotoxins, pesticides, heavy metals, persistent organic pollutants, pathogenic bacteria, and viruses; (ii) apply a standardized evaluation framework that explicitly codes each study for field-readiness limitations (e.g., need for magnetic separation, centrifugation, precise temperature control); and (iii) synthesize cross-cutting insights to expose the central tension between analytical sophistication and operational simplicity, a crucial but underappreciated challenge in the translation of lab innovations to real-world environmental monitoring.

**Figure 1:**
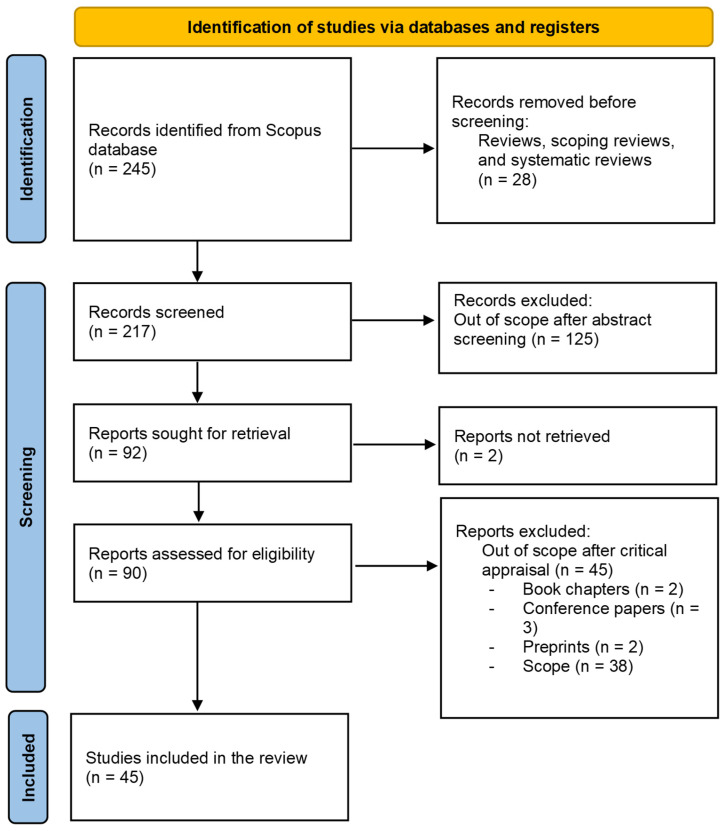
PRISMA 2020 flow diagram for the publications identified for the review (2016–2025).

In this systematic review, the Scopus database was searched between 2016 and 2025 for publications associated with following search keywords: “personal glucose meter”, “PGM”, “heavy metals”, “pesticides”, “viruses”, “enzymes”, “DNA”, “antibiotics”, “antibodies”, “inorganic substances”, “mycotoxins”, and “persistent inorganic compounds”. The analysis of search results indicates that the number of publications on PGM has increased more than 200% in last five years. The Prisma 2020 flow diagram associated with number of included studies is presented in Figure 1.

This systematic review is organized as follows: Section 2 describes the determination of chemical ecotoxicants using PGMs including antibiotics, mycotoxins, pesticides, heavy metals, inorganic substances and persistent organic pollutants. Section 3 describes the determination of biological ecotoxicants using PGMs including bacterial cells and DNA, and viruses. Section 4 describes the comparative assessment of key biosensing strategies and systematic comparison of environmental ecotoxicants. This paper discusses various ecotoxicant detection strategies using PGM for in situ analysis.

## 2. PGM-Based Determination of Chemical Ecotoxicants in Environment

### 2.1. Antibiotics

**Figure 2:**
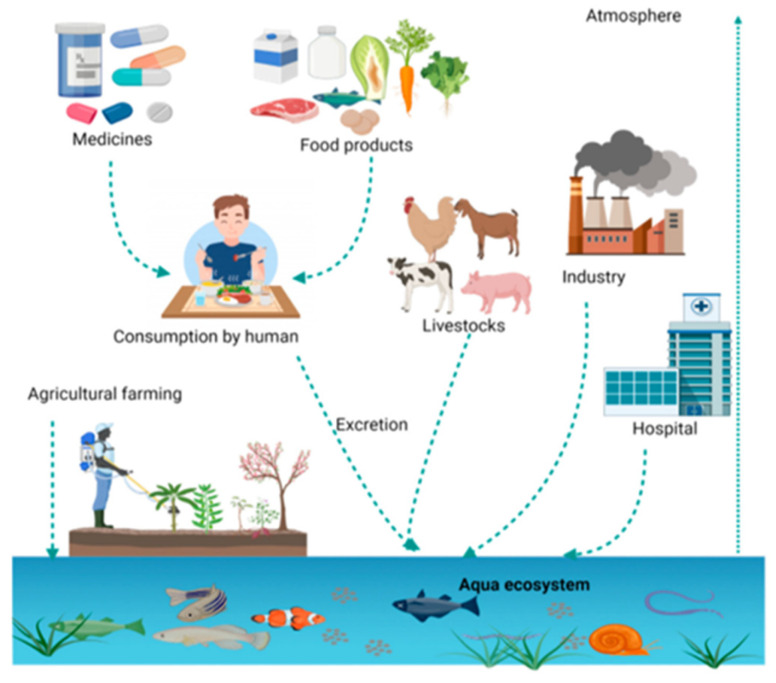
Sources of environmental pollution by antibiotics (reproduced with permission from [16], copyright 2021 MDPI).

Antibiotics are an extensive group of ecotoxicants of both natural and synthetic origin, which are actively used in healthcare and animal husbandry to combat pathogenic microorganisms. Their widespread use has led to a significant impact on the environment and human health [12,13,14,15]. The ways in which antibiotic residues may enter the environment are shown in Figure 2 [16].

Thus, one example of the effect of antibiotics on the aquatic ecosystem is the ability of antibiotics to change the structure of microbes and thus destroy many types of beneficial microorganisms, and can also lead to the translocation of antibiotics through the food chain into the human body [12]. Once in the human body, antibiotics cause a number of negative consequences, including an increase in allergic reactions, disruption of the natural microbiome and a decrease in the effectiveness of antibacterial therapy due to the development of resistance in pathogens [17]. In addition, the persistence of antibiotics in the environment creates additional risks, since these substances are characterized by a long half-life and the ability to accumulate in biotic and abiotic components of ecosystems. Consequently, the widespread use of various antibiotics in the environment poses a complex problem that requires fast, sensitive, affordable and portable methods for their detection [18,19]. In this context, the use of PGMs seems to be a highly promising approach for creating a new generation of biosensors. The main approach is to use specific bioreceptors (antibodies or aptamers) labeled with an enzyme (invertase or glucose oxidase). When such a conjugate binds to the target antibiotic, an enzyme is released that catalyzes the oxidation or hydrolysis of glucose, the concentration of which is directly measured using a portable glucose meter [20]. This allows for operational monitoring in the field, bypassing complex laboratory equipment to create an early warning and control system. To date, sensor platforms for detecting antibiotics using PGM have already been created and successfully tested, which, to simplify reading, will be conditionally divided into biosensors using antibodies, aptamers and microorganisms.

#### 2.1.1. Antibody-Based PGM Biosensors

Biosensors using antibodies as bioreceptors have recently demonstrated high efficiency in detecting antibiotics [21,22], since their use allows for high detection selectivity even in complex matrices, high sensitivity and reproducibility of results. Despite this, since 2015, a limited number of papers have been presented in the literature where antibodies are used in combination with PGM for the quantitative determination of antibiotics (Table 1). An example of such a symbiosis is the work [23], in which an invertase capable of catalyzing sucrose hydrolysis to glucose was conjugated with monoclonal antibodies to norfloxacin (a fluoroquinolone antibiotic, NOR) using colloidal gold. In addition, magnetic particles (MB) with immobilized NOR-BSA were used, which entered into a competitive interaction with free norfloxacin for binding sites on signaling probes. The use of MBs in many studies is explained by the significant simplification of the analysis procedure due to the possibility of efficient and rapid separation of unrelated reagents under the influence of a magnetic field. The developed biosensor was used to quantify NOR via a PGM-based competitive assay in animal products, demonstrating a wide linear range 0.5–500 ng·mL^−1^ with an LOD 0.5 ng·mL^−1^ [23]. Despite the high sensitivity, the developed technique is characterized by significant multi-stage and long analysis time, which limits its use for express control. An additional limitation is the complexity of synthesis and the potential instability of such a biosensor over time, since enzymes and antibodies are sensitive to long-term storage conditions.

#### 2.1.2. Aptamer-Based PGM Biosensors

**Figure 3:**
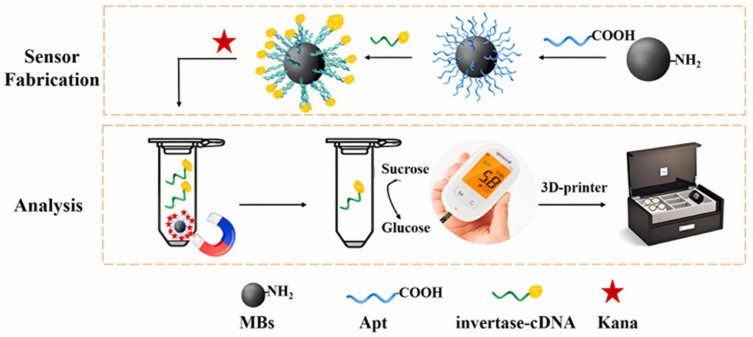
Graphical representation of the stages of analysis of kanamycin in water by PGM (reproduced with permission from Ref. [26], copyright 2025 Elsevier B.V.).

Due to their advantages, such as easy modification and cheap production, aptamers have become a promising alternative to antibodies in the development of biosensors for the detection of various antibiotics [24]. The high specificity of aptamers makes it possible to use them in biosensors as recognition elements capable of detecting antibiotics at the nanomolar level. Over the past decade, several types of aptamer-based biosensors using PGM have been developed for the quantification of ampicillin [25], ofloxacin [20] and kanamycin [26] using PGM. The general detection principle in these works involves five stages: (1) preparation and immobilization of the bioconjugate; (2) competitive binding of the antibiotic in the sample; (3) magnetic separation; (4) enzymatic reaction; and (5) determination of the concentration of glucose formed using PGM, which is proportional to the content of the target antibiotic in the sample. For example, the authors of the [26] immobilized a carboxylic kanamycin aptamer on Fe_3_O_4_ magnetic nanoparticles. Complementary DNA labeled with the enzyme invertase was attached to this complex, which in turn is capable of hydrolyzing sucrose to glucose. The resulting magnetic aptasensor made it possible to indirectly detect kanamycin in river water with a PGM, incorporating 3D printing technology (Figure 3).

The authors achieved low sensitivity (LOD 0.28 nM with a wide LDR of 1–200 nM), high specificity and stability (25 days). However, when selecting optimal conditions, it was found that the reaction requires a temperature of 37 °C and a pH of 7.4 for the hydrolysis of sucrose to glucose. However, these specific requirements present a challenge for developing biosensors for on-site detection, as they necessitate equipping the PGM with additional thermostating and buffer control systems. This, in turn, complicates the device design and increases its cost. In addition, the effect of hydrolysis time was not studied, even though the reliability and accuracy of the analysis depend directly on the reaction’s completeness.

#### 2.1.3. Whole-Cell Microbial PGM Biosensors

Another interesting alternative to enzyme biosensors for determining antibiotics with PGM is the registration of metabolic glucose consumption by bacteria [27]. In this method, the conversion of the analyte into a glucose signal is carried out by the bacteria’s metabolism, rather than by enzymatic transduction or nanocarriers. An example of this approach is the work presented in [28], which describes a direct method for detecting the residual antibiotic enrofloxacin in water and milk based on monitoring the metabolic activity of *E. coli* using PGM. The antibiotic’s antibacterial effect suppressed bacterial growth, which reduced glucose consumption. Consequently, the glucose concentration in the medium increased, leading to a PGM signal that was inversely proportional to the antibiotic concentration. This system achieved an enrofloxacin LOD of 5 ng·mL^−1^, which is significantly lower than the maximum residual limit (MRL). Despite its simplicity and low cost, this analysis method has several critical disadvantages due to the biological nature of the sensor element. The main limitations include the long analysis time (3–5 h), insufficient selectivity (reaction to any antibacterial drugs) and the difficulty of maintaining stable activity of the biological component in the field. In addition, Kwon D. et al. propose an alternative approach to the determination of enrofloxacin in real objects based on the use of paper glucose test strips [28]. The colorimetric method of analysis allows to visually assess the concentration of the antibiotic by changing the color of the indicator strip: with an increase in the glucose content in the sample, the color changes from yellow to darker green. However, this method requires further improvement, as it only allows for a semi-quantitative estimation of enrofloxacin concentration based on color intensity, without providing precise quantitative data.

### 2.2. Mycotoxins

Mycotoxins produced by fungi of the genera *Aspergillus*, *Penicillium*, and *Fusarium* are persistent environmental pollutants. Dangerous representatives of this class of ecotoxicants include aflatoxins, ochratoxins, fumonisins and trichothecenes, which have carcinogenic, teratogenic and immunosuppressive effects on human and animal health [29,30,31]. Amid growing demand for rapid diagnostics, traditional mycotoxin analysis methods face fundamental limitations, such as the need for sophisticated equipment and qualified operators [32]. This review therefore examines test systems that use PGM for mycotoxin detection, a method that combines the accuracy of electrochemical detection with the device’s inherent accessibility and ease of use.

#### 2.2.1. Enzyme Biosensors Based on Antibodies

Aflatoxin M1 is a hydroxylated metabolite of aflatoxin B1, which is formed in the liver of animals and excreted in milk when contaminated feed is consumed. The toxin does not break down during pasteurization, ultrapasteurization and other types of heat treatment, making it difficult to eliminate from the food chain and requires strict control at all stages of dairy production. Therefore, a portable device for rapid screening is especially important for farmers, as it allows for the timely detection of milk contamination, preventing economic losses and mitigating risks to consumer health. The PGM-based immunosensor developed in [33] solves this problem, offering a sensitive and specific method for detecting aflatoxin M1 with a LOD of 27 ppt. In this work, specific polyclonal antibodies (IgGMS-M1) with high avidity for aflatoxin M1 were produced and conjugated with invertase. After incubation of the resulting conjugate with milk samples, the aflatoxin M1 content was determined by measuring the glucose concentration formed during sucrose hydrolysis using a standard glucose meter. This development offers a promising solution for milk safety screening in the field in about 2 h without expensive equipment; however, the study did not establish a linear quantification range for the mycotoxin [33]. Furthermore, critical analytical characteristics such as selectivity, stability, and reproducibility were not investigated. It is also important to note the use of invertase, which requires strictly defined conditions for its operation. The enzyme reaction of sucrose hydrolysis critically depends on the temperature and pH of the medium, which in real conditions is difficult to control and maintain stable without special additional equipment.

#### 2.2.2. Enzyme Biosensors Based on Aptamers

**Figure 4:**
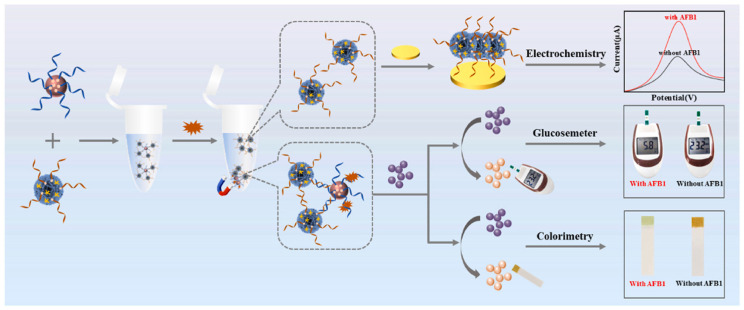
Principle of the electrochemistry–glucosemeter–smartphone integrated multi-mode biosensor to detect aflatoxin B1 (reproduced with permission from Ref. [35], copyright 2025 Elsevier B.V.).

The integration of aptameric biosensors with PGM makes it possible to convert the specific binding of mycotoxins into a universal glucose measurement signal. Thus, in [34] an anti-ochratoxin A (OTA) aptamer immobilized on the sensor surface was used, which was hybridized with complementary biotinylated probe for creating a recognition system. Biotinylated invertase was then introduced and immobilized on the probe via streptavidin, providing signal amplification due to the conversion of sucrose into glucose. Various mass crops, including wheat, corn, barley, rice, coffee, among others, can be easily contaminated with OTA during cultivation, harvesting, storage, and transportation. Under optimal conditions for glucose production (pH 5.5, 0.5 M sucrose, 55 °C, 50 min incubation), the biosensor achieved sensitive OTA detection in the range of 0.5–10 ng·mL^−1^ with a detection limit of 0.45 ng·mL^−1^ [34]. Although the biosensor demonstrated good practical application for analyzing real rice samples, with OTA recoveries of 88–103%, its use in the field has limitations. Conducting a multi-stage analysis protocol in the field is difficult because it requires the use of a thermostat, pH meter and other specialized equipment, which hinders on-site testing at the point of sampling. Another example is the work presented in [35], where a multimode biosensor for aflatoxin B1 was developed. This biosensor combines electrochemical detection, PGM and smartphone-based colorimetric quantification. Figure 4 shows the biosensor preparation scheme and the principle of detecting aflatoxin B1 in real objects using the example of wine and buckwheat flour.

This biosensor is based on the integration of multifunctional SA-Cu_3_(PO_4_)_2_ hybrid nanoflowers, acting as a carrier for biotinylated invertase and DNA probes, which provide three complementary detection modes: electrochemical (due to the redox activity of copper), glucometric (through enzymatic glucose generation) and colorimetric (visualization using test strips). This synergy enabled excellent analytical characteristics; however, the multi-stage conjugate production and the environmental sensitivity of invertase could compromise the reproducibility of results in field applications. Since 2015, biosensors for the detection of mycotoxins using PGM without magnetic separation have been developed, but, unfortunately, comparable sensitivity and reliability of operation in real matrices without this stage are not achieved. Moreover, the lack of data in most studies on the behavior of sensors in the presence of complex matrix components and during long-term storage does not allow an objective assessment of their stability in environmental conditions.

### 2.3. Pesticides

The various types of pesticides used in modern agriculture are necessary to control pests and increase yields. After application, they do not disappear without a trace; their residues can persist in the soil, pollute groundwater and surface waters, and accumulate in organisms of non-target species, triggering a cascade of environmental violations [36]. Their impact on key parts of ecosystems is particularly dangerous: they cause the death of pollinating insects such as bees [37], disrupt the reproductive functions of birds and fish [38], and also reduce the biodiversity of soil microflora and fauna, leading to long-term ecosystem degradation and reduced sustainability [36]. This devastating environmental impact poses direct risks to human health, as humans become the final link in the food chain by consuming food and water containing pesticide residues [39,40]. Chronic exposure to even low doses of these substances has been linked to the development of serious diseases, including various forms of cancer, neurodegenerative disorders, and endocrine disorders [41]. Thus, environmental pollution with pesticides forms a vicious cycle in which short-term agricultural benefits turn into long-term threats to environmental stability and human health. including traditional techniques like high-performance liquid chromatography with tandem mass spectrometry [42], and fluorimetry [43], as well as various (bio)sensor platforms based on electrochemical analysis [44,45,46]. Using PGM as a base platform transforms complex laboratory analyses into portable, quantitative, and inexpensive screening tools. Such devices open up the possibility for mass monitoring of pesticides directly in the field, which is a key step towards ensuring food security and preventing health risks. This approach is still under active development, as evidenced by the relatively small but growing number of publications on PGM-based pesticide quantification (Table 1).

#### 2.3.1. Antibody-Based PGM Biosensors

As mentioned above, antibodies have a high specificity for target molecules, which makes them ideal recognition elements in biosensors. For example, the immunosensor for carbendazim developed in [47] uses PGM as a portable reader has been developed. Carbendazim is a fungicide from the benzimidazole class, widely used in agriculture to control fungal diseases of plants. The method was based on multi-stage signal amplification. This involved: (1) using gold nanoparticles to immobilize secondary antibodies and DNA; (2) enzymatic chain elongation with terminal deoxynucleotidyl transferase (TdT); and (3) the final enzymatic generation of glucose by invertase for detection by the glucose meter. The developed biosensor was used to quantify carbendazim in samples of citrus fruits, canned citrus fruits and cabbage with an LOD 0.37 ng·mL^−1^. Despite the high sensitivity, the method has potential selectivity problems. It responds to thiophanate-methyl and benomyl, which decompose to form carbendazim, potentially causing false positives. In addition, the method critically depends on maintaining the activity of several biological components (antibodies, enzymes, DNA) during its long, multi-stage process. This necessitates strict control over analysis and storage conditions, which compromises its robustness and practical utility.

#### 2.3.2. Aptamer-Based PGM Biosensors

Non-enzymatic sensor systems, devoid of the key disadvantages of their enzyme analogs—such as low stability and sensitivity to environmental conditions—present significant potential for developing new PGM-based biosensors. One such option is the use of nanozymes (functionalized nanomaterials with inherent enzymatic activity), which have high catalytic activity over a wide range of pH and temperatures. Modern nanozymes are synthesized from various materials [48], but metal–organic frameworks (MOFs) attract the most research interest due to their high density of bioinspired active centers. Thus, the authors of [49] used a Co-MOF-based bioinspired nanozyme in combination with an aptamer-switching approach to detect acetamiprid in agricultural soil, water source and foodstuffs. The principle of operation of the biosensor is based on the fact that the binding of acetamiprid to the aptamer triggers the disassembly of the T_1_/T_3_ DNA duplex. The released T_1_ sequence hybridizes with the complementary T_2_ sequence on the Au@Co-MOF/T_2_ surface, which leads to the immobilization of the nanozyme at the sensing interface. The subsequent catalytic oxidation of glucose by immobilized nanozyme enables quantification of acetamiprid via PGM [49]. The developed method demonstrates a linear response in the range of 1–200 nM with a detection limit of 0.42 nM, which meets the World Health Organization (WHO) requirements for monitoring pesticide residues. However, the complex, multi-stage preparation and analysis procedure is a drawback for field use, with a total analysis time of 230 min: T1/T3 hybridization (70 min); aptamer-acetamiprid binding (50 min); T1/T2 hybridization (70 min); and the catalytic reaction (40 min). In addition, the synthesis of such a nanozyme significantly increases the cost of analysis.

#### 2.3.3. Enzyme Inhibition-Based PGM Biosensors

**Figure 5:**
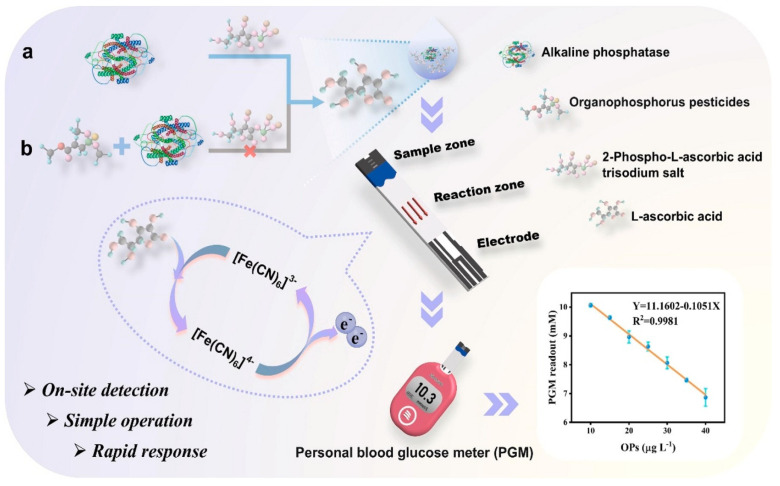
Schematic illustration of organophosphorus pesticide (OPs) detection using a PGM (**a**) In the absence of OPs, high ALP activity leads to significant AA production resulting in a higher PGM signal (**b**) In the presence of OPs the inhibition of ALP reduces AA production, leading to a corresponding decrease in the PGM readout(reproduced with permission fr1om Ref. [51], copyright 2025 Elsevier B.V.).

Traditionally, bioconjugates are widely used in the design of biosensors—complex structures that include specific recognition receptors such as aptamers, antibodies, or DNA. However, biosensors based on enzyme systems that do not require bioconjugates are particularly valuable for creating accessible platforms for the rapid detection of ecotoxicants in food and the environment. When a PGM is used, this approach greatly simplifies the quantification process. It also reduces cost and increases speed for analyzing pesticides in real-world samples. In 2025, two papers were published [50,51], that proposed a unified strategy for portable pesticide detection using PGM. Despite the different choice of enzymes, the fundamental principle is as follows: (1) the enzyme catalyzes the hydrolysis of a specific substrate to form a product with pronounced reducing properties, and (2) the resulting reducing agent reacts with an electronic mediator [Fe(CN)_6_]^3−^ in a standard PGM test strip, which leads to the generation of a measurable current recorded by a PGM. For example, the study by Yang, Jing et al. used the enzyme alkaline phosphatase to hydrolyze ascorbic acid-2-phosphate trisodium salt, producing ascorbic acid as a reducing agent (Figure 5) [51].

This method allows for fast (20 min) and sensitive (LOD 7.55 µg·L^−1^) dimethoate determination in cucumbers, Pak choi, pears and apples. However, the method has disadvantages, including limited selectivity and susceptibility to interference from reducing agents present in sample matrices. Alkaline phosphatase can be inhibited not only by dimethoate but also by other organophosphate pesticides; therefore, the method is better suited for monitoring the total contamination level of this pesticide class. In addition, natural reducing agents in the analyzed fruit and vegetable samples can create an interfering background signal by directly interacting with the electronic mediator of the test strip. Thus, this method is valuable for primary screening but requires further improvement for accurate identification and quantification.

### 2.4. Heavy Metals

Heavy metals are metallic elements characterized by a high density and high atomic weight. Although many heavy metals occur naturally, some enter the biosphere through anthropogenic activities [52]. Heavy metals are characterized by high toxicity, environmental persistence, and the ability to accumulate in humans and animals [53]. The pollution of terrestrial and aquatic ecosystems with heavy metals is a serious environmental problem, creating an urgent need for rapid detection methods.

#### 2.4.1. Nanomaterial-Assisted PGM Sensors

**Figure 6:**
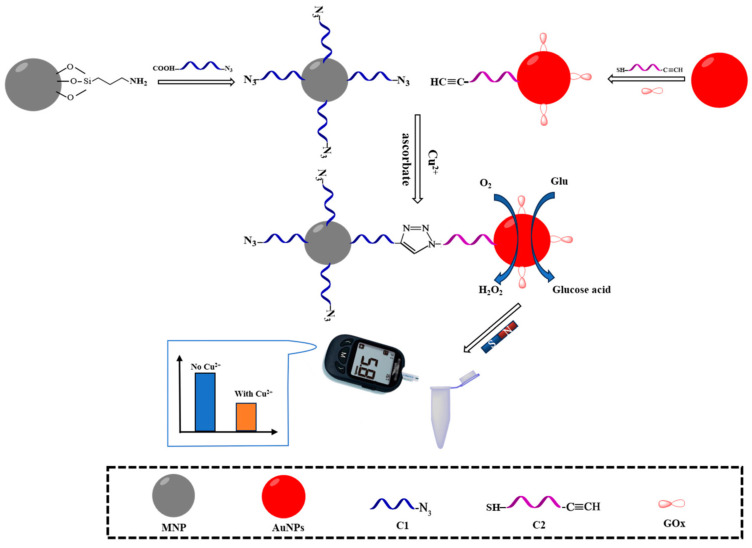
Schematic diagram of Cu^2+^ detection using a PGM (reproduced with permission from [55], copyright 2024 MDPI).

Copper, as one of the most common heavy metals, plays an important role in numerous biological processes and exists mainly as the Cu^2+^ ion. In the human body, copper is essential for the normal functioning of the nervous and immune systems. However, excess Cu^2+^ prevents bone regeneration, leads to a decrease in bone marrow tissue, accumulates in the liver, causes oxidative stress and has a neurotoxic effect on the brain and nervous system [54]. A detection system for copper was developed by the authors [55], who created a portable method for determining Cu^2+^ using a PGM in combination with a Cu^+^-catalyzed click reaction between alkyne and azide groups. The method was based on the use of magnetic Fe_3_O_4_ nanoparticles (MNPs) functionalized with the azide-containing oligonucleotide sequence C1 (MNPs-NH_2_-C1) and gold nanoparticles (AuNPs) modified with glucose oxidase (GOx) and alkyne DNA C2 (AuNPs-GOx-C2). The detection principle involved the reduction of Cu^2+^ to Cu^+^ by sodium ascorbate. The resulting Cu^+^ then catalyzed a “click” reaction, coupling the azide group on the MNPs with the alkyne group on the AuNPs. This led to the binding of the AuNPs-GOx-C2 complex to the MNPs-NH_2_-C1 surface. The subsequent magnetic separation made it possible to isolate the conjugates formed. Glucose oxidase immobilized on AuNPs catalyzed the oxidation of glucose to gluconic acid and hydrogen peroxide (H_2_O_2_), which led to a decrease in glucose concentration in solution, quantified using PGM. The PGM signal was inversely proportional to the Cu^2+^ concentration because higher Cu^2+^ levels led to more AuNPs-GOx-C2 binding to the magnetic particles, thereby reducing the glucose concentration. The authors determined the following optimal conditions: a 45 min incubation for the AuNPs-GOx-C2 to bind to the MNPs-NH2-C1 at room temperature, and a 50 min enzymatic reaction with 20 mM glucose at 37 °C and pH 7.0 (Figure 6). The method demonstrated a linear detection range of Cu^2+^ copper ions from 0.05 to 10.00 µM with a detection limit of 0.03 µM. The authors tested the manufactured biosensor on real tap water samples using the standard addition method. The recovery ratio ranged from 92.30% to 113.33%. According to the article, this method is highly selective and sensitive in water samples. The disadvantages of the developed system include the multi-stage analysis and the use of reducing agents like ascorbate, which can interfere with other sample components.

Silver ions are another target of study. Silver can accumulate in the body, interacting with elements like sulfur and selenium; chronic exposure can lead to argyrosis, a condition characterized by blue-gray skin discoloration. For example, the authors [56] developed a portable method based on the inhibition of invertase enzyme activity in magnetic crosslinked invertase aggregates (MCLIA). The principle involved the specific binding of Ag^+^ ions to invertase’s active site, decreasing its catalytic activity in the hydrolysis of sucrose to glucose. The decrease in the concentration of glucose produced, recorded by the glucose meter, was proportional to the concentration of Ag^+^. The signal was detected using an Accu-Chek Active glucose meter after a 45 min incubation of the sucrose hydrolysis reaction at 35 °C. The method demonstrates a linear range from 5 to 70 µM with a detection limit of 4.6 µM. The authors tested the method by adding known amounts of Ag^+^ to model solutions. The MCLIA selectively detected Ag^+^ in the presence of other metal ions (Ni^2+^, Cu^2+^, Co^2+^, Zn^2+^), confirming its potential for analyzing complex matrices.

#### 2.4.2. Nucleic Acid-Based PGM Biosensors

Chunchuan Gu et al. [57] developed a different system: a portable, single biosensor platform for the sequential detection of copper ions (Cu^2+^) and pyrophosphate (PPi). The platform used a DNA enzyme system with Fe_3_O_4_ MNPs and a PGM for signal readout. The core principle centered on an “off-on-off” signal mechanism modulated by the target analytes. The sensor structure was formed by modifying the surface of Fe_3_O_4_ MNPs synthesized by the hydrothermal method with a SiO_2_ shell. The obtained Fe_3_O_4_-SiO_2_ nanoparticles were treated with APTES (triethoxysilane) to obtain Fe_3_O_4_-NH_2_ and modified with streptavidin (SA) in the presence of glutaraldehyde (25%). Simultaneously, the authors synthesized a “Cu^2+^-dependent DNAzyme-invertase conjugate” (Cu-sub), where invertase was linked to a DNA sequence cleavable by a Cu^2+^-dependent DNAzyme. Next, Fe_3_O_4_-SA MNPs were bound to DNA in the Cu sub-invertase by a specific reaction between streptavidin and biotin. In the initial state, after magnetic separation and sucrose injection (1 M), the system generated a low background signal on the glucose meter. When Cu^2+^ ions were introduced into the system after 2 h at 43 °C, specific cleavage of Cu-sub DNA occurred under the action of a DNA enzyme (Cu-Enz), which led to the release of invertase into solution and the conversion of sucrose to glucose. The glucose level was recorded by PGM after 40 min at room temperature. A significant increase in the signal led to the “switching on” of the system (“off-on” switching). For subsequent detection of PPi, pyrophosphate was introduced into the same system, which formed a stable Cu^2+^-PPi complex after 2 h at 43 °C. As a result, after magnetic separation, the invertase remained in the solution, catalyzing glucose production and leading to a high signal. The subsequent addition of PPi sequestered the Cu^2+^, preventing DNA cleavage and causing a sharp signal decrease (the “on-off” switch) recorded 40 min after sucrose introduction. The developed method demonstrated a linear detection range of Cu^2+^ and PPi of 0.01–5 and 0.5–10 µM with detection limits of 10 nM and 500 nM, respectively. The authors tested the manufactured biosensor by determining Cu^2+^ in tap water samples using the standard addition method. The recovery rate ranged from 96.8% to 116%. Human urine from Hangzhou Cancer Hospital was selected as a sample for the determination of PPi. The PPi recovery rate ranged from 96.6% to 109.6%. The good selectivity of the developed sensor is one of the advantages of this work. Disadvantages include a complex, multi-stage sensor preparation, a long total analysis time, and non-standard conditions requiring specialized equipment. Furthermore, the detection limit for copper ions may be insufficient for stringent environmental regulations.

**Figure 7:**
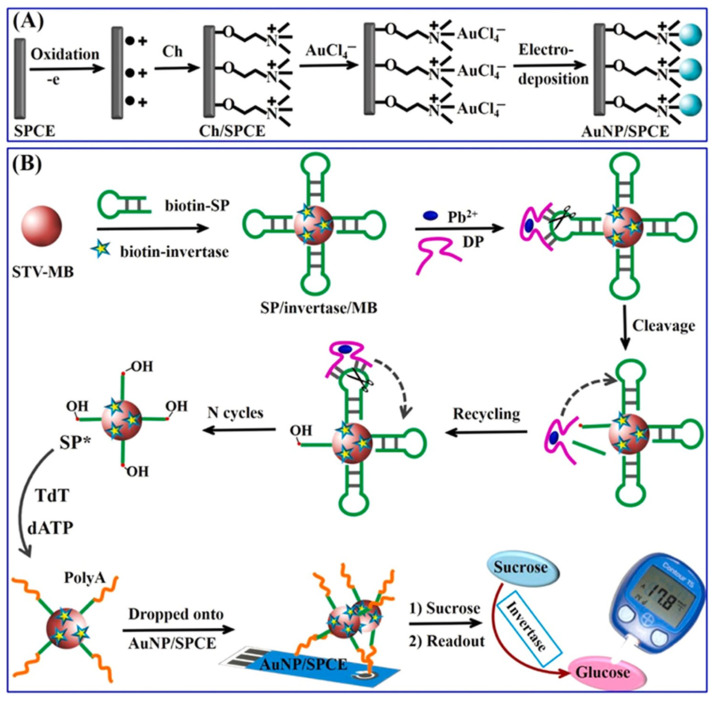
(**A**) AuNP/SPCE fabrication procedure; (**B**) Schematic representation of a TdT-mediated sensor for sensitive and POC detection of Pb^2+^ (reproduced with permission from Ref. [59], copyright 2024 Elsevier B.V.).

Lead is another common and toxic heavy metal. Like copper, ingested lead ions accumulate in the body, exerting severe toxicological effects. Lead exposure adversely affects the human reproductive system, impairs fetal neural stem cell development, causes neurotoxicity impacting behavioral and cognitive functions, and disrupts the cardiovascular system [58]. For lead detection, the authors [59] developed a method using cascaded signal amplification, which combined a DNAzyme cyclic reaction with terminal deoxynucleotidyltransferase (TdT) polymerization, and detected the signal with a PGM (Figure 7). The method relied on the Pb^2+^-dependent activation of a DNAzyme immobilized on magnetic particles. The activated DNAzyme catalyzed the cyclic cleavage of a substrate DNA, generating numerous 3′-OH ends. These ends served as centers for TdT, which catalyzes the synthesis of extended polyadenine (polyA) sequences. The amplification products were immobilized on the surface of a screen-printed carbon electrode modified with gold nanoparticles (AuNPs/SPCE) due to the high affinity interaction of polyA with gold, providing specific binding of magnetic particles conjugated with invertase. The immobilized invertase then catalyzed the hydrolysis of sucrose to glucose, and the resulting glucose concentration was quantified with the PGM. The enzymatic reaction of sucrose hydrolysis was carried out for 20 min at room temperature. The method achieved a low detection limit of 0.6 pM and a wide linear range from 0.002 nM to 2000 nM. The authors tested their methodology on real samples of tap and river water and obtained recovery values in the ranges 98.2–103.2% and 97.4–104.9%, respectively. The method’s advantages include high sensitivity and effective signal amplification from the combined DNAzyme cleavage and TdT-mediated polyA synthesis. Disadvantages include a complex, multi-stage protocol and a long total analysis time exceeding 190 min.

Cadmium is another toxic heavy metal. Its accumulation in human tissues induces oxidative stress, leading to cellular damage. This process can provoke the development of lung, kidney, prostate and pancreatic cancers [60]. To detect cadmium ions, the authors [61] developed a method using an aptamer for specific Cd^2+^ recognition, which triggers a cascade signal amplification via exonuclease III (Exo III) recycling. The system included magnetic particles (MBs) pre-coated with SA, then sequentially functionalized with DNA seed (DNA1) and hairpin DNA (DNA2), forming the SA-MBs-DNA1-DNA2 complex. Separately from the obtained complex, the authors bound Cd^2+^ to the aptamer (DNA3) in the DNA3-DNA4 duplex. SA-MBs-DNA1-DNA2 and Exo III were then added to this mixture. A pre-synthesized DNA invertase conjugate (DNA5-invertase) was added to the resulting system. After magnetic separation and washing, the immobilized invertase catalyzed sucrose hydrolysis, and the resulting glucose was quantified by PGM. The signal was directly proportional to the Cd^2+^ concentration. For the PGM measurement, 5 µL of the final solution was applied to a test strip and read after 30 s. At the same time, the color of the control window of the test strip changed from yellow to green. The method demonstrated a linear detection range of Cd^2+^ from 20 pM to 200 nM with a detection limit of 5 pM. The authors tested the finished biosensor on real samples of water (lake, river, pond).

**Figure 8:**
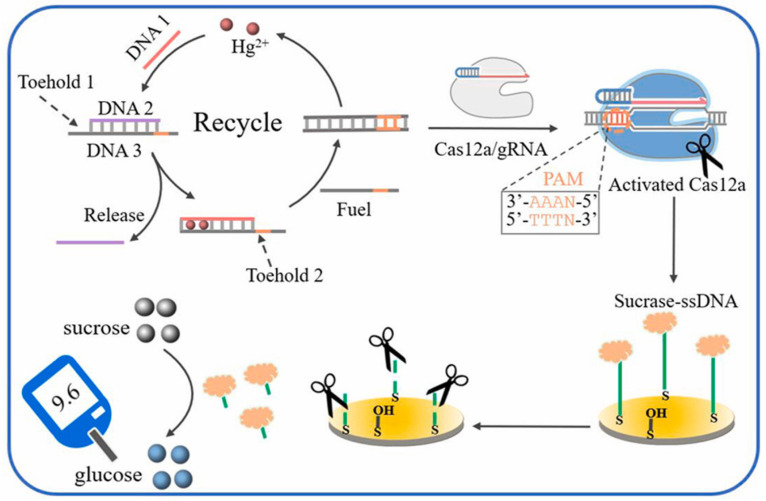
Schematic diagram of a portable biosensor for the quantitative determination of Hg^2+^ based on TSDR and CRISPR-Cas12a (reproduced with permission from Ref. [63], copyright 2023 Elsevier B.V.).

Mercury is one of the most toxic heavy metals for humans. Mercury ions (Hg^2+^) affect red blood cells and other orgsans, disrupting their function and damaging the cardiovascular system [62]. The authors [63] developed a highly sensitive method for portable Hg^2+^ detection by combining a cascading DNA strand displacement reaction (activated by T-Hg^2+^-T complexes) with the CRISPR-Cas12a system, using a PGM for glucose signal detection (Figure 8). The presence of Hg^2+^ initiated cascading substitution of DNA strands, leading to the generation of multiple double-stranded DNA that activated the trans-nuclease activity of Cas12a. Activated Cas12a then cleaved a DNA-invertase conjugate immobilized on an electrode, releasing invertase into the solution. The free invertase catalyzed the hydrolysis of sucrose to glucose, which was then measured by the PGM. The enzymatic sucrose hydrolysis was conducted for 30 min at 37 °C before glucose concentration was measured with the PGM. The method achieved a wide linear range from 100 fM to 10 nM with a detection limit of 40 fM. The authors tested the technique on real samples of tap, river and wastewater. The method showed high accuracy and reproducibility: when adding known concentrations of Hg^2+^, recovery values in the range from 97.0% to 100.8% were obtained, which confirms the reliability for monitoring Hg^2+^ in the environment.

Methods also exist for the simultaneous detection of multiple heavy metals. For example, the authors [64] developed a method for detecting heavy metal ions using a cell-free protein synthesis system and a personal glucose meter. The method uses a genetic circuit where the expression of an *E. coli* invertase gene is controlled by promoters responsive to metal-sensitive transcription factors—CadC for Cd^2+^ and Pb^2+^, and SmtB for Zn^2+^. When a target metal ion binds to the repressor (CadC or SmtB), it dissociates from the DNA operator, triggering transcription of invertase mRNA and its translation into functional enzyme within the cell-free system. The synthesized invertase catalyzed the hydrolysis of sucrose to glucose, and the resulting concentration was measured using a PGM. A key condition for minimizing the background signal from transcriptional leakage was a short, 10 min cell-free synthesis incubation at 30 °C. After synthesis, 15 µL of the reaction mixture was incubated with an equal volume of 200 mM sucrose in phosphate buffer for 10 min at 37 °C. The reaction was then stopped by heating to 90 °C for 5 min, centrifuged, and the glucose concentration in the supernatant was measured with the PGM. The authors detected cadmium, lead, and zinc ions in the micromolar concentration range. However, the article does not describe testing the technique on real water samples [64].

### 2.5. Inorganic Substances

**Figure 9:**
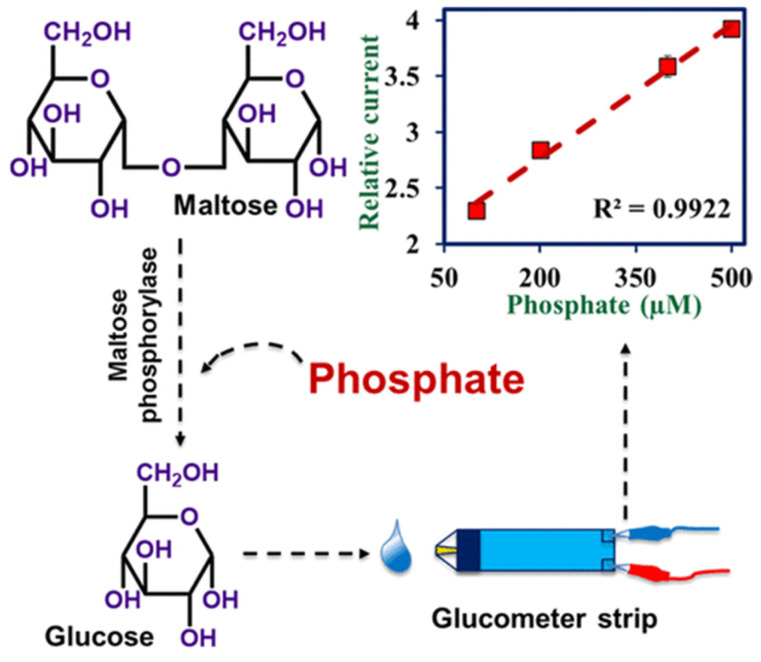
Determination of orthophosphate using test strips of a PGM and a potentiostat (reproduced with permission from Ref. [65], copyright 2022 ACS).

Beyond heavy metals, there is a growing need for portable, rapid methods to detect other inorganic substances that can also disrupt ecosystems. For example, the authors [65] developed an enzymatic method for the portable determination of orthophosphate (PO_4_^3−^) in water using glucose meter test strips connected to a mini-potentiostat. The method was based on a reaction catalyzed by maltose phosphorylase (MP), where orthophosphate acted as a substrate. The enzyme catalyzed the breakdown of maltose to form glucose and glucose-1-phosphate; the amount of glucose produced was stoichiometric with the PO_4_^3−^ concentration. The resulting glucose was quantified amperometrically using an Accu-Check Aviva test strip containing pyrroloquinoline quinone-dependent glucose dehydrogenase (PQQ-GDH) and mediators. On the test strip, glucose was oxidized to gluconolactone, generating a current proportional to the glucose concentration that was measured by the potentiostat. For analysis, a mixture was prepared containing 50 µL of MP (16 U/mL), 100 µL of sample, and 50 µL of maltose (3.2 mM) in 0.1 M citrate buffer (pH 5.75). This mixture was incubated at 30 °C for 135 min. The reaction was stopped by heating to 100 °C for 15 min to denature the enzyme. After centrifugation, 80 µL of supernatant was applied to a test strip. Amperometric measurement was performed at 450 mV, with the current averaged between 3 and 20 s. The method demonstrated a detection limit of 1.45 µM (0.1 ppm) with a dynamic range from 10 µM to 3 mM. The use of a mini-potentiostat provided significantly higher sensitivity than a standard PGM, which has a detection limit ≥600 µM for glucose. The authors validated the method on tap and river water samples spiked with PO_4_^3−^ (Figure 9).

**Figure 10:**
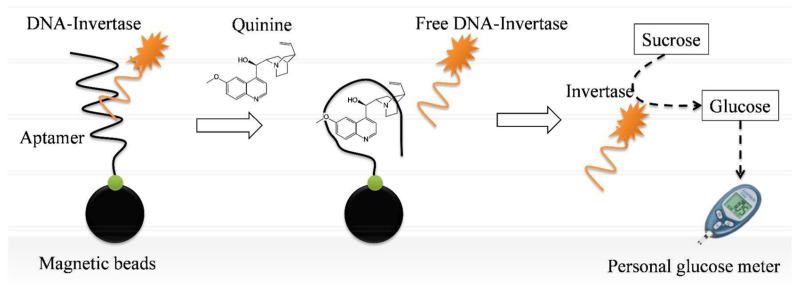
Scheme of the MBs-aptamer-invertase protocol for the determination of quinine in water using a PGM (reproduced with permission from Ref. [66], copyright 2018 RSC).

Another example of a non-glucose analyte detection using a PGM is presented in [66]. The article presents an aptamer method for the determination of quinine in water, including in treated wastewater, using PGM (Figure 10). The method is based on the competitive binding of quinine to an aptamer immobilized on magnetic beads (MBs). At the first stage, a biosensor structure was formed on the surface of magnetic particles functionalized with streptavidin (SA). A biotinylated, quinine-specific aptamer (MNS-4.1) was immobilized on the particles and hybridized with a complementary oligonucleotide conjugated to invertase (DNA-invertase). Upon introduction of a sample containing quinine, the target bound specifically to the aptamer. This caused the DNA-invertase conjugate to be released into the solution. After magnetic separation, the supernatant containing the free invertase was incubated with sucrose. The glucose produced was then quantified using the PGM. The full analysis took about 40 min. The method demonstrated a linear detection range of quinine from 0.1 to 2 µM, with a detection limit of 0.13 µM in pure water and 0.32 µM in treated wastewater.

### 2.6. Persistent Organic Pollutants

PCB77 (3,3′,4,4′-tetrachlorobiphenyl), as one of the most toxic dioxin-like polychlorinated biphenyls, not only poses a danger to ecosystems but also threatens public health. The article [67] describes the use of a portable PGM for the sensitive detection of PCB77. For this purpose, a smart DNA-hydrogel was prepared that is degraded by the CRISPR/Cas system. To analyze PCB77, the PCB77 aptamer was first hybridized with its complementary cDNA strand. In the presence of PCB77, the aptamer binds its target, causing dehybridization and the release of cDNA. This cDNA then primes a rolling circle amplification (RCA) reaction. The RCA product activates the Cas12a enzyme. Activated Cas12a then cleaves the single-stranded DNA linkers within the DNA-hydrogel, releasing encapsulated invertase. The free invertase catalyzes sucrose hydrolysis, producing glucose for quantification by PGM. The combination of RCA and CRISPR/Cas system amplification enables highly sensitive PCB77 detection with the PGM. The authors conclude that the developed biosensor is suitable for online monitoring, being characterized by low cost and ease of operation. However, to implement this approach in point-of-care analysis, additional equipment for the CRISPR/Cas system will be required.

**Table 1:** PGM-based determination of the chemical Ecotoxicants in the environment.

Sensor Type/ Enzyme	Conjugate Type	Format of Assay	Stages of Analysis	LDR/ LOD	Required Equipment	Temperature Sensitivity	POC Readiness	Sensor Characteristics		Performance Metrices	Target Object	Ref.
								Adv.	Disadv.			
Antibiotics												
Enzymatic/ glucose oxidase	MOF based enzyme-DNA conjugate	aptamer-based competitive assay	- incubation of MB-Apt/Ce-MOF-GOx-cDNA with OFL; - magnetic separation; - enzymatic reaction (glucose is oxidized by glucose oxidase)	50 pg·mL^−1^–500 ng·mL^−1^/ 40 pg·mL^−1^	W	Ambient	No	A, B, D, E	N, Q, R	(1) 14 d. storage at 4 °C 96%; (2) 5 interferents tested; (4) RSD 1.1–5%; (5) 99.5–110%	ofloxacin in water, milk and chicken	[20]
Enzymatic/ invertase	IgG-invertase	antibody-based competitive assay	- incubation of sensing probe (NOR-OVA-MBs) with signal probes (colloid gold-invertase-Ab) and test sample; - magnetic separation; - enzymatic reaction (sucrose is hydrolyzed by invertase)	0.5–500 ng·mL^−1^/ 0.5 ng·mL^−1^	V, W, X	Controlled 37 °C (binding), 55 °C (hydrolysis), 25 °C	No	A, B, D, E	N, Q, R	(2) 18 interferents tested; (4) RSD 8.61–30.27%; (5) 73.14–137.37%	norfloxacin in milk and pork	[23]
Enzymatic/ invertase	biotinylated invertase conjugate	aptamer-based competitive assay	- the biotinylated aptamer is fixed to the MB surface via a streptavidin bridge; - saturation of the aptamer with the invertase-ampicillin conjugate; - magnetic separation; - enzymatic reaction (sucrose is hydrolyzed by invertase)	2.5 × 10^−10^–1.0 × 10^−7^ mol·L^−1^/ 2.5 × 10^−10^ mol·L^−1^	V, W, X	Controlled 25 °C (binding), 50 °C (hydrolysis)	No	A, B, D, E	N, Q, R	(2) 4 interferents tested; (3) RSD 4.8–5.6%; (4) RSD 4.2–4.9%; (5) 83.57–126.49%	ampicillin in milk	[25]
Enzymatic/ invertase	MBs-Apt-Inv-cDNA	aptamer-based competitive assay	- production of invertase-cDNA modified MBs-Apt; - incubation of MBs-Apt-invertase-cDNA with kanamycin solution; - magnetic separation; - enzymatic reaction (sucrose is hydrolyzed by invertase)	1–200 nM/ 0.28 nM	V, W, X	Controlled 37 °C (hydrolysis)	No	A, B, D, E	N, Q, R	(1) 25 d. 82% retention; (2) 5 interferents tested; (4) RSD 2.35%; (5) 95.6–110%	kanamycin in water	[26]
Non-enzymatic	–	suppression of the growth of *E. coli* bacteria by an antibiotic	- incubation of a mixture of glucose, LB, *E. coli* and real object under the 37 °C; - measuring the decrease in glucose consumption by *E. coli* due to inhibition of bacterial growth by an antibiotic	–/ 5 ng·mL^−1^	X	Controlled 37 °C (incubation)	No	A, B, C, D, E, F, G	–	(2) 4 interferents tested; (3) 4.24–4.95%	enrofloxacin in water and milk	[28]
Enzymatic/ invertase	β-cyclodextrin/ invertase polymer bioconjugate	antibody-free sandwich assay	- production of sandwich-type complexes by mixing CAP with m-MIP and bioconjugate EV–Au–β-CD/INT; - magnetic separation; - enzymatic reaction (sucrose is hydrolyzed by invertase)	0.5–50 ng·mL^−1^/ 0.16 ng·mL^−1^	V, W, X	Controlled 40 °C	No	A, B, E	N, O, Q, R	(1) 21 d. 90% retention; (2) 3 interferents tested; (3) 2.7–4.7%; (4) 2.1–4.5%; (5) 86.2–102.2%	chloramphenicol in fish and pork samples	[68]
**Pesticides**												
Enzymatic/ alkaline phosphatase, deoxynucleotidyltransferase	sAb/DNA-AuNPs	antibody-based competitive assay with DNA-mediated signal amplification	- incubation of the analyzed sample with monoclonal antibody of carbendazim; - incubation of the resulting mixture with secondary Ab/DNA-AuNPs; - incubation with addition of deoxynucleotidyltransferase and biotin-16-dCTP; - signal amplification via the enzyme label (streptavidin-alkaline phosphatase); - enzymatic reaction (glucose-1-phosphate is hydrolyzed by alkaline phosphatase)	0–100 ng·mL^−1^/ 0.37 ng·mL^−1^	X	Controlled 37 °C	No	A, B, E, F, G	N, O	(2) 14 interferents tested; (3) RSD 5.16%; (4) 5.8–12.8% RSD; (5) 70.4–109.4%	carbendazim in canned citrus, citrus and cabbage	[47]
Non-enzymatic	aptamer-functionalized bioinspired nanozyme	aptamer-switching assay	- incubation of an acetamiprid solution with a pre-prepared surface modified with AuNPs and T1/T3 sequences to form an acetamiprid-T3 complex; - addition of the Au@Co-MOF/T2 probe and its incubation for T1 and T2 sequence hybridization; - catalytic reaction (oxidation of glucose by the Co-MOF complex)	1–200 nM/ 0.42 nM	X	Controlled 37 °C (incubation)	No	A, B, D, E, F, G	N	(2) 5 interferents tested; (3) RSD 3.8–6.2%; (5) 82.6–113.9%	acetamiprid in agricultural soil, water source and foodstuffs	[49]
Enzymatic/cholinesterase	–	direct enzymatic assay	- incubation of the sample with the cholinesterase enzyme; - enzymatic reaction (acetylthiocholine is hydrolyzed by cholinesterase to produce thiocholine)	0.05–1 ppm/ mevinphos—0.138 ppm carbofuran—0.113 ppm	None	Ambient 25 °C	Yes	A, C, D, E, F, G	M	(2) 2 pesticides, inter-species interferences from distinct inhibition patterns; (5) 90.8–136.1%	mevinphos and carbofuran in cabbage	[50]
Enzymatic/ alkaline phosphatase	–	direct enzymatic assay	- incubation of the analyzed sample with alkaline phosphatase; - enzymatic reaction (L-ascorbic acid-2-phosphate trisodium salt is hydrolyzed by alkaline phosphatase to produce ascorbic acid)	10–40 µg·L^−1^/ 7.55 µg·L^−1^	None	Ambient	Yes	A, C, D, E, F, G	M	(1) 12 d.; (2) multiple organochlorines and neonicotinoids; (4) RSD 0.23–2.75%; (5) 96.1–108.1%	dimethoate in cucumber, Pak choi, pear and apple	[51]
Enzymatic/acetylcholinesterase	–	direct enzymatic assay	- incubation of the analyzed sample with the acetylcholinesterase enzyme; - enzymatic reaction (acetylthiocholine is hydrolyzed by acetylcholinesterase to produce thiocholine)	0–30 µg·L^−1^/ 5 µg·L^−1^	None	Ambient	No	C, D, E, F, G	L, M	(5) 95.0–103.4%	paraoxon in apple and cucumber	[69]
**Mycotoxins**												
Enzymatic/ invertase	IgG-invertase	antibody-based competitive assay	- incubation of the analyzed sample with IgGMS-M1-invertase; - enzymatic reaction (sucrose is hydrolyzed by invertase)	–/ 27 ppt	V	Ambient	No	A, E, F	N, R	–	aflatoxin M1 in whole milk	[33]
Enzymatic/ invertase	Apt/Biotin-cDNA	aptamer-based competitive displacement assay	- obtaining double-stranded Apt/Biotin-cDNA products; - immobilization of the complex on the AuNPs modified SPCE; - incubation of the product with streptavidin and biotylated invertase; - enzymatic reaction (sucrose is hydrolyzed by invertase)	0.5–10 ng·mL^−1^/ 0.45 ng·mL^−1^	V, X	Controlled 55 °C	No	A, B, D, E, F	N, R	(2) tested for <5 interferents; (3) RSD <3.39%; (4) 88.0–103.0%	ochratoxin A in rice	[34]
Enzymatic/ invertase	Apt/SA-MBs	aptamer-based competitive assay	- immobilization of biotinylated aptamer onto SA-MBs and beanding with SA-Cu_3_(PO_4_)_2_ hybrid nanoflowers-cDNA-biotinylated invertase; - incubation of the complex with the analyzed solution containing aflatoxin B1; - magnetic separation; - enzymatic reaction (sucrose is hydrolyzed by invertase)	0.01–50 ng·mL^−1^/ 5.4 pg·mL^−1^	W, X	Controlled 37 °C (binding) 55 °C (hydrolysis)	No	A, B, E, G	N, O, Q	(1) 21 d. 80% retention; (2) 6 interferents tested; (3) RSD 1.7%; (4) 1.4–4.1%; (5) 97.7–105.1%	aflatoxin B1 in wine and buckwheat flour	[35]
Non- enzymatic	biotin-conjugated anti-AFB_1_ antibody	antibody sandwich assay	- incubation of antibodies to AFB_1_ with the analyzed solution; - immobilization of biotinylated anti-AFB_1_ antibody, streptavidin, and biotinylated glucose-encapsulated liposomes; - hydrolysis of the liposome by 1X PBS-Tween 20 buffer	0.001–10 ng·mL^−1^/ 0.6 pg·mL^−1^	V	Ambient 25 °C	No	A, B, E, F	N, O, R	(2) 5 interferents tested, interference is observed for AFB_2_; (5) 94–112%	aflatoxin B1 in peanuts and new-born cattle serum	[70]
Enzymatic/ invertase	biotin-OTA aptamer	aptamer-based competitive assay	- immobilization of biotinylated aptamer on SA-MBs and hybridization with DNA-invertase signaling probe; - real sample addition and incubation; - magnetic separation; - enzymatic reaction (sucrose is hydrolyzed by invertase)	0–100 µg·L^−1^/ 3.66 µg·L^−1^	V, W	Ambient	No	A, E	M, N, O, Q, R	(2) 2 interferents tested	ochratoxin A in red wine	[71]
Enzymatic/ invertase	biotin-substrate strand- invertase	aptamer-based competitive assay	- immobilization of biotin−aptamer probe−DNAzyme with biotin−substrate strand−invertase on MBs; - reaction of the analyzed sample with the resulting system; - magnetic separation; - enzymatic reaction (sucrose is hydrolyzed by invertase)	1–300 pg·mL^−1^/ 0.88 pg·mL^−1^	V, W	Ambient	No	A, B, E	N, O, Q, R	(2) 9 interferents tested; (5) 93.1–108.3%	ochratoxin A in red wine	[72]
**Heavy metals**												
Enzymatic/glucose oxidase	MNPs-NH2-C1/AuNPs-GOx-C2	Cu^+^-catalyzed click chemistry on magnetic platform	- preparation of multiple conjugates; - conducting a click-chemistry reaction in the presence of Cu^2+^, ascorbate and THPTA; - magnetic separation and washing; - enzymatic reaction of GOx with glucose; - detection of the remaining glucose on the PGM	0.05–10.00 μM/ 0.03 μM	V, W, X	Controlled 37 °C	No	A, E	M, N, O, Q, R	(2) 7 interferents tested; (3) RSD 0.8–1.2%; (5) 92.3–113.33%	Cu^2+^ in real tap samples	[55]
Enzymatic/ invertase	MCLIA (magnetic nanoparticles with APTES and immobilized invertase aggregates)	Enzyme inhibition assay	- MCLIA incubation with a sample containing Ag^+^; - magnetic separation and washing; - enzymatic reaction with the addition of a sucrose; - measurement of glucose concentration using a PGM	5–70 μM/4.6 μM	W, X	Controlled 35 °C	No	A, G	M, N, O, P, Q	(2) 4 interferents tested	Ag^+^	[56]
Enzymatic/ invertase	DNA-invertase conjugate	DNAzyme cleavage with magnetic separation	- sensor preparation; - addition of Cu^2+^ or PPi ions; - magnetic separation; - sucrose is hydrolyzed by invertase. - glucose measured with a PGM	Cu^2+^: 0.01–5 μM PPi: 0.5–10 μM/ Cu^2+^: 10 nM PPi: 500 nM	V, W, X	Controlled 43 °C	No	A, B, E	N, O, Q, R	(1) 14 d. 91.2%; (2) 7 interferents tested; (5) 96.6–116.0%	Cu^2+^ in tap water; PPi in urine	[57]
Enzymatic/ invertase	SP/invertase/MB conjugate	TdT-mediated DNA extension amplification	- substrate DNA (SP) and the enzyme invertase are immobilized on MB; - Pb^2+^ detection and cleavage; - signal amplification by the TdT extension by polyA sequences; - polyA is captured on the gold electrode; - enzymatic reaction of invertase with sucrose	2–2000 nM/ 0.6 pM	V, W, X	Controlled 37 °C	No	A, B, E	N, O, Q, R	(1) 30 d. storage at 4 °C 94.9%; (2) 10 metal and organic interferents tested; (3) RSD 5.1%; (4) RSD 3.2%; (5) 97.4–104.9%	Pb^2+^ in river and tap water	[59]
Enzymatic/ invertase	DNA5-invertase conjugate	Aptamer-based with Exo III amplification	- DNA1-DNA2 probe assembly; - Cd^2+^ detected with addition of DNA3-DNA4; - Exo III cleavage; - magnetic separation; - invertase hydrolyzes sucrose into glucose; - glucose is quantified using a PGM	20 pM –200 nM/ 5 pM	V, W	Ambient	No	A, B, E	N, O, Q, R	(2) 9 interferents tested	Cd^2+^ in lake, river, and pond water	[61]
Enzymatic/ sucrase	Sucrase-ssDNA probe	cascade DNA chain substitution with CRISPR-Cas12a	- Hg^2+^ is incubated with DNA complexes (DNA 1, DNA 2/DNA 3, fuel) to trigger TSDR and produce Cas12a activators; - freed sucrase produces glucose; - Glucose measurement with a PGM	100 fM –10 nM/ 40 fM	X	Controlled 37 °C	No	A, B, E, F, G	N, O	(1) 15 d. 97.8% retention; (2) 7 interferents tested; (4) RSD 3.1%; (5) 97.0–100.8%	Hg^2+^ in water samples	[63]
Enzymatic/ invertase Non-enzymatic/Nano-Glo Luciferase (NLuc)	Cell-free genetic circuit	heavy metal-responsive protein synthesis	**Invertase**: - cell-free protein synthesis; - sucrose hydrolysis by synthesized invertase; - glucose measurement with PGM test strip **Nano-Glo Luciferase** - cell-free protein synthesis; - dilution of reaction mixture; - luminescence measurement after adding substrate	–/ Cd^2+^: 6.5 µM Pb^2+^: 2.2 µM Zn^2+^: 2.4 µM	V, X	Controlled 37 °C	No	A, B, F	N, O, P, R	(2) 3 interferents tested	Cd^2+^, Pb^2+^, Zn^2+^	[64]
**Inorganic substances**												
Enzymatic/Maltose Phosphorylase	Direct enzyme	enzymatic cleavage of maltose	- mix sample with MP and maltose; - incubate at 30 °C for long time; - reaction Termination (100 °C for 15 min to denature the enzyme); - Glucose measurement with potentiostat	10 μM–3 mM/ 1.45 μM	V, X, Y	Controlled 37 °C	No	A, B, C, D, E, F	R	(2) 7 interferents tested	PO_4_^3−^ in water	[65]
Enzymatic/ invertase	DNA-Invertase Conjugate	competitive aptamer-based assay	- chemical conjugation of thiol-DNA and invertase; - biotinylated aptamer immobilization and hybridization with the DNA-invertase conjugate; - magnetic separation; - adding sucrose to the supernatant and incubating for invertase to produce glucose; - PGM-based glucose detection	0.1–2 μM/ 0.13 μM (pure water) 0.32 μM (wastewater)	V, W	Ambient 25 °C	No	A, E	M, N, O, Q, R	(1) 14 d. storage at 4 °C; (2) 4 interferents tested	Quinine in water and reclaimed wastewater	[66]
**Persistent organic pollutants**												
Enzymatic/ invertase	invertase-encapsulated DNA hydrogel	CRISPR/Cas12a derived biosensing platform	- preparation of the invertase-encapsulated DNA hydrogel; - pre-amplification via RCA process; - the activation of Cas12a; - dissociation of DNA hydrogels; - enzymatic reaction (sucrose is hydrolyzed by invertase)	1.0·10^−4^–1.0 µg·L^−1^/3.2·10^−5^ µg·L^−1^	X	Controlled 37 °C	No	A, B, E, F, G	N, O	(1) 14 d.; (2) 7 interferents tested for PCB77; (3) RSD 3.7%; (5) 95.0–105.0%	PCB77 in drinking water	[67]

**Required Equipment. V**—Centrifuge, **W**—Magnetic rack, **X**—Thermostat, **Y**—Potentiostat, **Z**—PCR cycler. **Sensor Characteristics Advantages:** A—Low LOD, B—Wide LDR, C—Easy conjugate preparation steps, D—Low cost of conjugate preparation, E—Validated matrix effects, F—Magnetic separation is not required, G—Centrifugation step is not required **Disadvantages:** L—High LOD, M—Narrow LDR, N—Complex conjugate preparation steps, O—High cost of conjugate preparation, P—Unvalidated matrix effects, Q—Magnetic separation is required, R—Centrifugation step is required **Performance Metrices:** (1)—Stability-d.: number of days, (2)—Specificity/Selectivity, (3)—Reproducibility (reported RSD), (4)—Repeatability (reported RSD), (5)—Recoveries.

**Figure 11:**
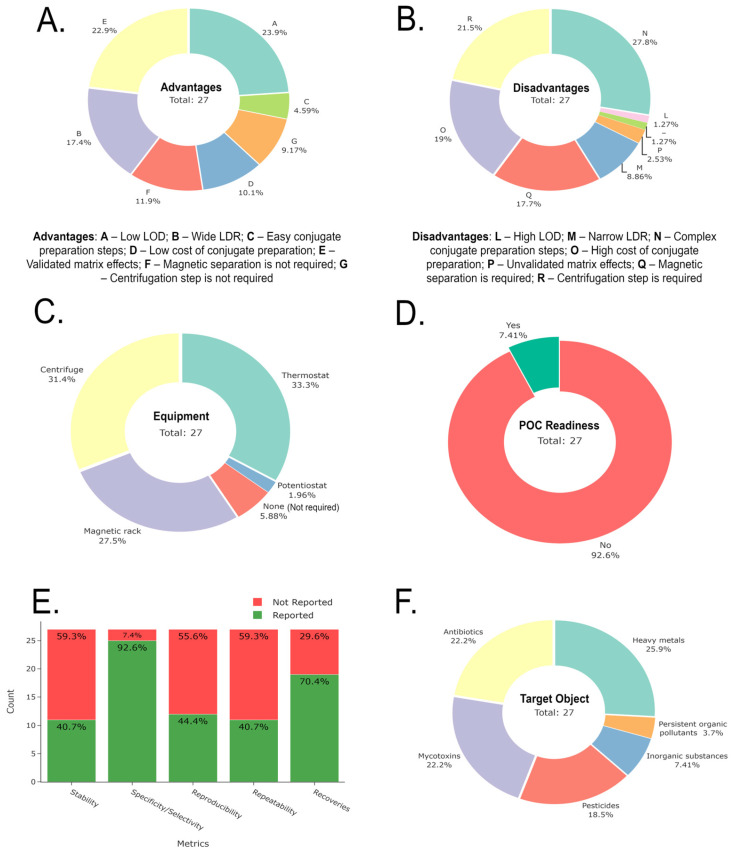
Percentage summary plots for the reviewed PGM-based sensors and biosensors for the determination of chemical ecotoxicants presented in Table 1. (**A**) individual advantage codes, (**B**) individual disadvantage codes, (**C**) requirement of additional equipment, (**D**) POC readiness, (**E**) performance metrices reported by the studies, and (**F**) type of chemical ecotoxicant determined by the studies.

Figure 11 illustrates a summary of the advantages, disadvantages, required equipment, POC readiness, and performance metrics for all PGM-based sensors and biosensors used for environmental ecotoxicants. Only 5.88% of PGM-based sensors and biosensors do not require specialized equipment. Furthermore, only 7.41% have achieved full POC readiness, representing a current limitation that needs improvement for future applications. The individual codes represent the relative percentage of each coded advantage or disadvantage, helping to identify the most prevalent features across the studies. In other words, individual percentages (feature prevalence) are the number of times individual code appears divided by the total code instance across all studies.

## 3. PGM-Based Determination of Biological Ecotoxicants in Environment

### 3.1. Bacterial Cells and DNA

Pathogenic microorganisms enter the soil, water, and air from secretions of sick people and animals, as well as from bacterial and viral carriers. Identification of pathogenic microbes helps to identify environmental contamination and to develop preventive measures to reduce bacterial contamination of various facilities. Microorganisms that enter the product during its production can cause food infections and poisoning. Prompt detection of pathogenic microorganisms helps to identify violations in food production technology, such as insufficient heat treatment or improper transportation and storage, which helps trace the food chain and confirm the source of infection. The identification and quantification of pathogens for clinical diagnosis, environmental monitoring, and food safety are critical to public health. Various sensitive, reliable and fast approaches, such as enzyme immunoassay (ELISA) [73], bacterial colony counting [74], PCR [75], fluorescence assays [76], electrochemical methods [77] and colorimetric strategies [78] are used to identify bacteria or their DNA/RNA. However, many of these methods are laborious and time-consuming. Furthermore, they require highly qualified specialists and the use of complex tools and expensive equipment. The search for alternative methods that are fast, portable, low-cost, and easy to use for pathogen detection is an urgent task. Paper-based microfluidic (PGM) devices possess these desirable properties. This section examines research articles that focus on the adaptation of PGMs for the control of bacterial pathogens in various samples, including biological fluids, environmental samples, and food (Table 2).

#### 3.1.1. Nucleic Acid-Based PGM Biosensors

Aptamers have excellent characteristics as bioreceptors and can be comparable or even superior to alternatives such as antibodies. The most important properties for the electroanalytical use of aptamers are: their production via in vitro synthesis and scalability [79]; their binding mechanism, which relies on non-covalent multipoint interactions including hydrogen bonds, electrostatic and hydrophobic interactions, π-π-stacking, and Van der Waals forces [80]; and their small size and linear structure, which allows for easy chemical modification at the 3′ or 5′ terminus. This capacity for modification is a key advantage. Aptamers are very stable because they can restore their native conformation after thermal treatment [81,82].

Aptamers are widely used as probes against bacterial molecules, and several studies have focused on the synthesis of aptamers using SELEX technology for live bacterial cell cultures targeting *Mycobacterium tuberculosis* [83], *Lactobacillus acidophilus* [84], *Staphylococcus aureus* [85], *Salmonella typhimurium* [86], and *Escherichia coli* O157:H7 [87]. Portable, inexpensive, and quantitative analysis of pathogens in the field and at home remains a key challenge in medical diagnostics, environmental monitoring, and food safety control. Over the past decades, extensive experience has been accumulated in using handheld portable devices for environmental, chemical, and medical applications

*Staphylococcus aureus* (*S. aureus*) is one of the most important bacterial pathogens, causing a range of diseases including sepsis, gastrointestinal infections, and food poisoning [88]. *S. aureus* is transmitted mainly through water and food. Rapid detection of *S. aureus* is essential to ensure public health and safety. Using the advantages of PGMs, the authors [89] developed an approach for the quantitative determination of *S. aureus*. The authors used aptamers as molecular recognition elements in combination with a signal amplification method based on sucrose hydrolysis by invertase to enable sensitive and selective detection. After binding the aptamer-labeled invertase to a bacterial cell, the authors proposed to separate the conjugate from unbound components by centrifugation followed by washing with phosphate-buffer solution (PBS). The authors note the need to maintain optimal conditions for invertase activity (pH 5.0 for 40 min). The authors also adjusted the pH of the medium from acidic to neutral (pH 7.0) with NaOH to measure the glucose analytical signal with a PGM. A linear relationship between the response and the logarithm of *S. aureus* concentration was achieved over a range of 1.2·10^5^ to 1.2·10^8^ CFU·mL^−1^, with a detection limit of 1.0·10^5^ CFU·mL^−1^. The advantages of the developed *S. aureus* aptasensor are its selectivity and the straightforward process for obtaining the aptamer-invertase conjugate. The disadvantages include the time-consuming separation step involving centrifugation. In addition, the enzymatic production of glucose is slow and cannot compete with the speed of methods like ELISA. The enzyme requires an elevated temperature of 50 °C, which makes it impossible to use this strategy in the POC format and in the field to monitor the level of bacteria in real samples.

**Figure 12:**
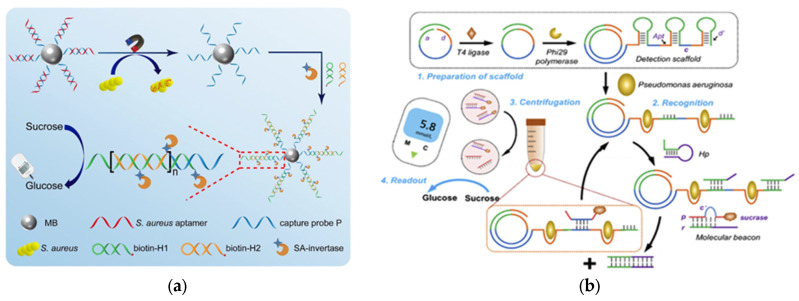
Schematic diagrams illustrating aptamer-based biosensor designs for detecting pathogenic bacteria with PGM (**a**) Detection mechanism for Staphylococcus aureus (**b**) Detection mechanism for Pseudomonas aeruginosa reproduced with permission from Ref. [90] 2021 and [94] 2023, copyright Elsevier B.V.).

The authors of [90] have developed a sensitive and highly selective magnetic aptasensor that uses a PGM for signal readout for the detection of *S. aureus*. The *S. aureus* aptamer was immobilized on a magnetic particle by hybridization with a capture probe. In the presence of *Staphylococcus aureus*, the aptamer dissociated from the magnetic particle. The released capture probe then hybridized with a biotinylated probe, triggering a DNA hybridization chain reaction (HCR) for signal amplification. Streptavidin-labeled invertase catalyzed glucose production from a sucrose substrate. Glucose concentration was determined using PGM, enabling portable quantitative detection of *S. aureus* (Figure 12a). The main advantage of the developed sensor is its high sensitivity, with a detection limit of 2 CFU·mL^−1^. Another advantage is its reliability, validated by comparing PGM results with a standard plate counting method. The disadvantages are the complex synthesis of the aptamer-based conjugates and the long enzymatic reaction time (60 min).

Magnetic separation with multiple washings and thermostating for the sucrose hydrolysis reaction makes the aptasensor unsuitable for use in the POC format. In addition, the authors do not take into account the effect of matrix glucose of the studied foods on the analytical signal of PGM. Nevertheless, the authors recommend this sensor for rapid portable detection of bacterial pathogens and suggest its potential for quality control in agricultural, food, and water safety applications.

Another work by the authors [91] is devoted to the development of a biosensor for the determination of the methicillin-resistant mecA *S. aureus* (MRSA) gene. Hairpin (H) probes are fixed on magnetic beads (MBs), enabling accurate target recognition and initiation of a target recycling process. The mecA gene binds to the stem-loop structure of the H probe, forming a duplex with a 3′ end that is recognized by Exonuclease III (Exo-III). Exo-III cleaves the probe, releasing the mecA gene and initiating the recycling of the target. The target recycling process initiates a HCR that aggregates sucrose-labeled probes, allowing for the conversion of sucrose to glucose, which is detected by PGM. This biosensor has shown promise for portable and sensitive detection of MRSA; however, it requires additional equipment, and the potential interference from endogenous glucose on PGM readings was not addressed.

*Pseudomonas aeruginosa* (*P. aeruginosa*) is a common Gram-negative bacterium that causes severe infections, especially in people with weakened immunity, and poses a huge threat to human health [92,93]. The authors of [94] have developed a highly sensitive sensor that combines low-speed centrifugation for isolating *P. aeruginosa* with PGM signal readout. To enhance the signal, a rolling circle amplification (RCA) strategy was used. The RCA product contains repeating sequences of aptamers for the specific capture of *P. aeruginosa*, enabling a wide dynamic range and a low detection limit of 36 CFU·mL^−1^. Other aptamers are labeled with sucrose, which is hydrolyzed to glucose in the supernatant (Figure 12b). In addition to its excellent sensitivity, this approach also demonstrates good selectivity for detecting *P. aeruginosa*, making it a promising tool for early detection. Despite the fact that RCA allows amplification with a minimum number of biological samples and avoids obtaining false positive results, the analysis requires additional equipment (a centrifuge and thermostat). This does not allow the sensor to be used to detect *P. aeruginosa* in the POC format in real analysis samples.

The work of the authors [95] is devoted to the development of a portable method for detecting *Salmonella typhimurium* in animal products. The invA gene of Salmonella was used as the biological recognition element. To ensure rapid on-site screening, the authors developed a dual-signal output method using CRISPR/Cas12a-RAA with lateral flow strips (LFS) and PGM for detection. The unique CRISPR/Cas12a-RAA system for the determination of the invA gene of Salmonella is capable of isothermal amplification due to recombinase polymerase (RPA) with a highly specific nucleic acid cleavage function, which improves the accuracy and sensitivity of the determination of the invA gene of Salmonella in animal products. However, despite its high sensitivity, this system is laborious and relatively expensive. In addition, the work did not consider the effect of matrix glucose of food products on the analytical signal of PGM.

**Figure 13:**
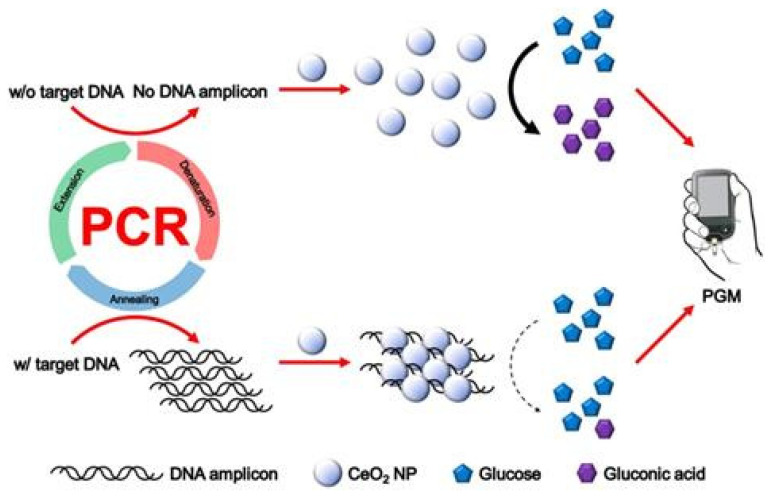
Schematic illustration of PGM-based label-free read-out of PCR amplification using glucose oxidase-like activity of CeO_2_ NPs (reproduced with permission from Ref. [96], copyright 2020 Ivyspring International).

The authors of [96] describe a label-free method for detecting *Escherichia coli* genomic DNA with a sensitivity of up to 10 copies·µL^−1^ using PGM. The method is based on the glucose oxidase-like activity of cerium oxide nanoparticles (CeO_2_ NPs). In the presence of the target DNA, PCR amplicons bind electrostatically to CeO_2_ NPs, causing their aggregation and inhibiting their catalytic activity. As a result, glucose is largely unoxidized, and glucose levels remain high. In the absence of target DNA, no amplicons are formed, and the CeO_2_ NPs effectively oxidize glucose, leading to a significant decrease in its concentration (Figure 13). The advantage of the developed approach is the absence of enzyme labels, which are prone to inactivation. However, the method requires PCR amplification, limiting its use to laboratory settings. The proposed sensor was tested only on model objects and did not consider the matrix effect of the components of real samples on the glucose signal measured by PGM.

#### 3.1.2. Antibody-Based PGM Biosensors

Immunoglobulins are still used as biosensor receptors for pathogenic bacteria. In the study by [97], the authors developed a sensitive method for detecting Salmonella using PGM. Clusters of MNPs functionalized with monoclonal antibodies were used to trap the bacteria. The MNP-Salmonella complexes were then magnetically separated from other components and conjugated with invertase that was functionalized with polyclonal antibodies before being dispersed in 0.5 M sucrose solution. After hydrolysis of sucrose to glucose and fructose, the glucose concentration was measured using PGM. To reduce the analysis time to 2 min, the authors recommend raising the temperature to 50 °C, which, however, complicates the procedure. The authors tested this method for the detection of Salmonella in milk with a detection limit of 10 CFU·mL^−1^ and suggested its potential for other samples. However, implementing this system for point-of-care (POC) use is difficult due to the risks of enzyme instability and the multi-step fabrication process.

In study [98], the authors developed a portable quantitative immunochromatographic sensor (ICS) using PGM as a reader for detecting *Escherichia coli* O157:H7. They prepared conjugates of Fe_3_O_4_ MNPs co-functionalized with invertase and antibodies (invertase-MNPs-IgG) to use as a probe. A pre-chromatographic paper strip, containing an absorbent pad, was impregnated with sucrose solution as an enzyme substrate. An innovative aspect of this approach is the combination of visualization via a brown magnetic nanoparticle stripe in the detection zone and quantification over a range of 2.08 × 10^5^ to 2.08 × 10^8^ CFU·mL^−1^ using PGM. The sensor showed selectivity for *E. coli* O157:H7, with no observed interference from other bacterial pathogens (*E. coli*, *S. aureus*, *C. freundii*, *C. sakazakii*, *L. monocytogenes*, *S. putrefaciens*, *B. subtilis*, *B. cereus*, *V. parahaemolyticus*). The authors recommend this approach as a universal analytical system for detecting a wide range of food pathogens and protein biomarkers, including applications in environmental monitoring.

**Figure 14:**
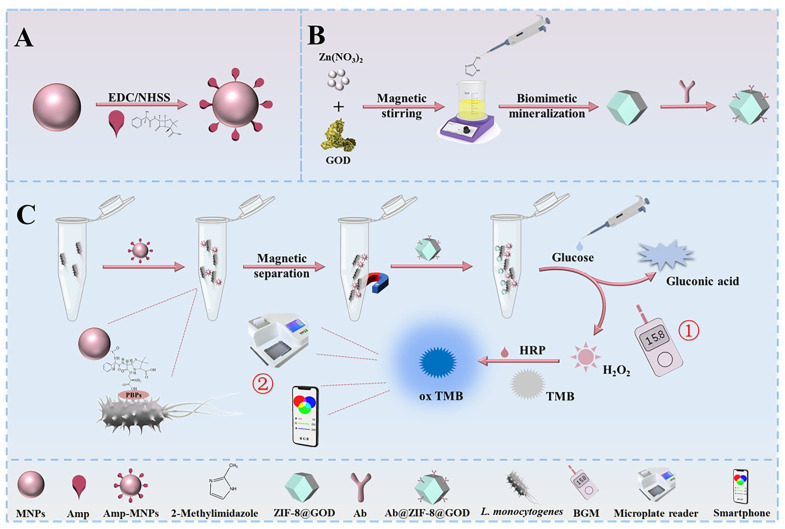
The illustration of preparation of Amp-MNPs (**A**) and GOD@ZIF-8@Ab (**B**) and dual-mode biosensor for on-situ analysis of *L. monocytogenes* (**C**) (reproduced with permission from Ref. [99], copyright 2023 Elsevier B.V.).

Environmental contamination by *Listeria monocytogenes* can pose a serious threat to human health, so there is an urgent need to develop sensitive on-site detection methods. In [99], the authors developed a field-deployable analysis combining magnetic separation with antibody-labeled, encapsulated glucose oxidase (GOD@ZIF-8@Ab) to capture and specifically identify *L. monocytogenes*. GOD catalyzes glucose oxidation, causing a change in the PGM signal (Figure 14). This biosensor showed good results in detecting *L. monocytogenes* in situ in lake water and juice samples, with a detection limit of 10^1^ CFU·mL^−1^ and a linear range from 10^1^ to 10^6^ CFU·mL^−1^. Thus, this locally deployable biosensor has promising potential for early screening of *L. monocytogenes* in environmental and food samples.

### 3.2. Viruses

Determining the presence of viruses in food and the environment helps minimize infection risks and develop preventive measures [100]. PGMs are being developed to detect viruses in environmental samples and food, offering inexpensive and accessible methods for in situ detection. This chapter provides a critical overview of the application of PGM for the detection of viral particles (proteins, DNA, RNA) in biofluids, environmental samples, and food products, as well as for the diagnosis of viral diseases (Table 2).

#### 3.2.1. Antibody-Based PGM Biosensors

Hepatitis B virus (HBV) infection continues to be a major public health problem and a leading cause of death worldwide. The most important diagnostic marker of this infection is hepatitis B virus surface antigen (HBsAg). The authors of [101] developed an inexpensive, portable and sensitive ELISA method with PGM signal measurement for the diagnosis of HBsAg. The ELISA system was created using a capture antibody (Ab1) against HBsAg, immobilized on streptavidin-coated iron oxide magnetic particles (SA-Fe_3_O_4_). A detection antibody (Ab2) against a different HBsAg epitope, and the enzyme glucoamylase, were immobilized on Al_2_O_3_ nanoparticles. After the formation of a sandwich immune complex, where HBsAg binds Ab1 on the magnetic particles and Ab2 on the Al_2_O_3_ nanoparticles—starch was converted into glucose by glucoamylase. The glucose concentration was then measured using PGM. Under optimal conditions, this assay showed detection limits of 0.3 ng·mL^−1^ and 0.4 ng·mL^−1^ for the HBsAg subtypes “ay” and “ad”, respectively. This study demonstrates that the developed method is comparable to commercial kits in terms of sensitivity, suitability for on-site analysis, specificity, cost, simplicity, portability, and reproducibility. While magnetic separation simplified washing and improved complex isolation, the key drawbacks are the multi-step conjugate fabrication process and the risk of enzyme inactivation during nanoparticle immobilization.

#### 3.2.2. NucleicAcid-Based PGM Biosensors

To date, PGM has been adapted and is popular for medical purposes, for example, for the quantitative determination of several protein biomarkers in blood serum, including antibodies to digoxin, thrombin, and antibodies to hepatitis C virus [102], cancer cells [103], cardiac troponin I [104], cancer biomarkers [105,106], atherosclerosis biomarkers [107], biomarkers of Alzheimer’s disease [108], screening for kinase-targeting drugs [109], and mRNA for disease diagnosis [103].

Since the first reports of a deadly respiratory disease in Wuhan, China, severe acute respiratory syndrome coronavirus 2 (SARS-CoV-2), the causative agent of COVID-19, has spread worldwide and led to the most devastating pandemic in more than a century. The main route of transmission of this virus is airborne, released from an infected person into the environment when coughing, sneezing, talking, or breathing. Another person becomes infected by inhaling the resulting aerosol. This pathogen has joined a growing number of emerging infectious diseases, including avian influenza, H1N1 influenza, human immunodeficiency virus (HIV), Ebola virus, Zika virus, Middle East respiratory syndrome coronavirus (MERS-CoV) and severe acute respiratory syndrome (SARS) coronavirus (SARS-CoV).

Currently, there are three generally accepted methods for diagnosing SARS-CoV-2 infection: (1) detection of viral RNA; (2) detection of viral proteins, such as the nucleocapsid (N) protein or the spike (S) glycoprotein; and (3) measurement of specific antibodies to viral proteins [110]. However, widespread testing for SARS-CoV-2 remains challenging due to limited clinical laboratory capacity, high costs, and the consequent high risk of disease spread, which leaves many populations without adequate screening [111].

Consequently, there remains an urgent need for accurate and cost-effective diagnostic tests that can be widely used. In this regard, a number of studies have focused on developing sensors for the detection of SARS-CoV-2 using PGM. The authors of [112] have developed an aptamer-based method for analyzing the SARS-CoV-2 antigen in saliva, which uses inexpensive reagents ($3.20 per test) and PGM. The authors use the catalytic properties of invertase and a competitive aptamer assay to convert antigen binding into an analytical glucose signal. Aptamers capable of selectively binding viral spike (S) or nucleocapsid (N) protein are pre-conjugated with invertase via a short oligonucleotide chain complementary to the aptamer’s binding domain. A biotinylated aptamer-oligo-invertase complex was immobilized on Fe_3_O_4_ NPs. In the presence of the antigen, the aptamer undergoes conformational changes, displacing the oligonucleotide-invertase conjugate, thus creating an antigen-sensitive switch. After magnetic separation, invertase hydrolyzes sucrose to glucose at a rate of 5 × 10^3^ mol·glucose·s^−1^, which enables a sensitive PGM signal. Despite the high selectivity and specificity of the sensor and the use of an inexpensive, easily modifiable aptamer, manufacturing of the sensor requires a complex procedure for obtaining an aptamer conjugate and its purification by magnetic separation. To obtain the product of the enzyme reaction, the system must be thermostatically controlled, which creates difficulties when using the sensor in the POC mode.

In [113], the authors propose a universal and quantitative platform for the analysis of nucleic acids based on PGM (PGM-NAAP). The SARS-CoV-2 nucleocapsid phosphoprotein (N gene) was tested as a model target, recognized by invertase-modified capture probes in a sandwich assay. Initially, the authors synthesized an oligonucleotide-invertase conjugate (Probe1) by covalent crosslinking. The Probe1-invertase conjugate, a biotin-modified Probe2, and the target SARS-CoV-2 DNA were combined. In the presence of the target nucleic acid, a “Probe1-invertase/target/Probe2” sandwich complex was formed via DNA hybridization. Then streptavidin-conjugated magnetic beads were added, capturing the sandwich structure. The complex was separated from unbound components by magnetic separation, washed, and incubated with sucrose; the resulting glucose was measured by PGM. Compared to existing methods, one of the advantages of the sensor is that it avoids interference from endogenous glucose, since the invertase-containing sandwich complex is separated from the sample. In addition, the sensor is stable for 20 days. Among the disadvantages are the need for a thermostat for the hybridization stage and the long sucrose hydrolysis time (1 h), which like the previous study, makes it difficult to use the sensor in the POC mode.

A similar test system was proposed by the authors of [114] based on competitive DNA hybridization. In the presence of the target DNA, competitive binding releases the DNA-invertase conjugate, which catalyzes the hydrolysis of sucrose to glucose, while other components remain on magnetic particles for easy magnetic separation. Unfortunately, this system has not been tested on real-world samples.

More recent work on DNA detection of viral particles relies on the CRISPR/Cas12a system as a target detection and transduction module. CRISPR/Cas12a is a powerful tool in biosensing that cleaves non-specific single-stranded DNA (ssDNA) after its guide RNA (crRNA) recognizes the target DNA [115].

For early diagnosis and control of the SARS-CoV-2 epidemic, the authors of [116] developed a test system based on clustered regularly interspaced short palindromic repeats (CRISPR)/Cas12a for portable, fast and sensitive detection of SARS-CoV-2. In this method, samples were pre-treated and amplified by reverse transcription-recombinase polymerase amplification (RT-RAA) under isothermal conditions. This process converts target viral RNA into cDNA for exponential amplification. Then, by combining the CRISPR Cas12a system and a glucose formation reaction, the virus signal was converted into a glucose signal quantifiable by PGM within seconds. The nucleocapsid protein gene was tested as a model target, and the detection sensitivity was 10 copies·µL^−1^, which meets clinical diagnostic needs.

Similarly, the authors of [67] described the use of PGM for sensitive detection of the SARS-CoV-2 N gene. For this purpose, a responsive DNA hydrogel was prepared, which is activated by the CRISPR/Cas system. In the presence of the target analyte, Cas12a is activated, cleaving single-stranded DNA linkers in the hydrogel and releasing invertase. The released invertase catalyzes sucrose hydrolysis, producing glucose measurable by PGM. Due to the amplification of RCA and the CRISPR/Cas system, a high sensitivity of N gene detection using PGM can be achieved. The authors conclude that the DNA hydrogel-invertase system is suitable for on-site monitoring, is low-cost, easy to operate, and applicable for SARS-CoV-2 analysis.

In a more recent paper, the authors of [117] propose an alternative technology for detecting SARS-CoV-2 RNA that is affordable, economical, portable, fast, and selective. The method is based on a cascade of enzymatic reactions without target amplification. The technology uses the CRISPR/LwaCas13a system, which non-specifically cleaves reporter RNAs (trans-cleavage) after specific target RNA recognition. In the proposed test system, the presence of the target SARS-CoV-2 RNA activates the trans-cleavage of CRISPR/Cas13a, generating 2′,3′-cyclic phosphate termini, which are converted to adenosine monophosphate (AMP) by polynucleotide kinase. AMP is subsequently converted to adenosine diphosphate (ADP) by myokinase; ADP is then used as a substrate in a cascade enzymatic reaction catalyzed by pyruvate kinase and hexokinase. The overall reaction continuously converts glucose to glucose-6-phosphate, decreasing the glucose concentration in proportion to the target RNA level, which is measured indirectly using PGM. The multi-enzyme sensor system for virus detection is expensive and carries the risks of inactivation of the latter, which significantly affects the detected PGM signal.

In recent works, one can also see a similar solid-phase invertase-based test system for detecting human papillomavirus (HPV) nucleic acids in biological samples [118]. A Cas/ILR/PGM platform for nucleic acid detection, which uses a solid immobilized invertase-labeled reporter (ILR), has been developed. The platform integrates nucleic acid detection using Cas12a or Cas13a. Invertase is immobilized on MBs using single-stranded RNA or DNA as linkers, which are cleaved upon CRISPR-Cas activation by target nucleic acids. As a result, active invertase is released, which converts sucrose into glucose in proportion to the amount of the target analyte. The HPV18-targeted test system is capable of detecting as few as 7 HPV18-positive cells among 7000, achieving a sensitivity of 95.8% and a specificity of 100% in the tested samples.

The authors of [119] proposed an innovative one-step strategy (FACER) for detecting nucleic acids without washing steps. The authors used the substrate-specific ability of flap endonuclease 1 (FEN1) to cleave DNA for its sensitive detection. FACER uses two specially designed probes that hybridize with the target DNA to form a flap structure cleaved by FEN1 to release adenosine monophosphate (AMP). AMP, as mentioned earlier, serves as a substrate in a cascade enzymatic reaction involving myokinase, pyruvate kinase, and hexokinase, which ultimately leads to a decrease in glucose concentration measured by PGM.

**Figure 15:**
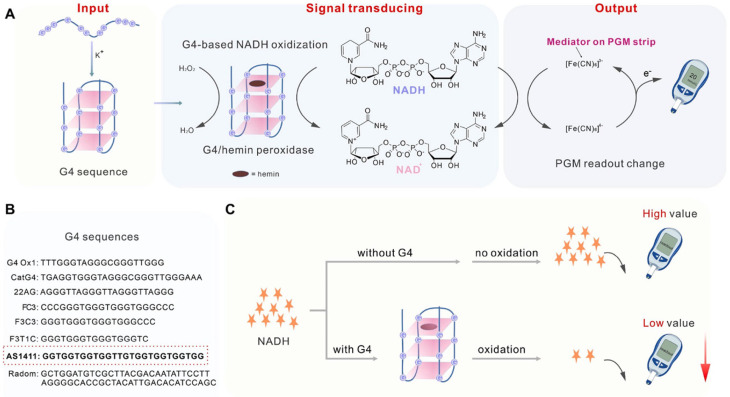
Linking the peroxidase-mimicking G4/hemin DNAzyme with NADH-based PGM readout. (**A**) Schematic illustration of G4-NADH-PGM system for target sensing, (**B**) Screening of different G4 sequences based on NADH oxidation reaction, The reaction contained 1 × G4 reaction buffer, 250 μM ATP, 1 μM of G4 sequence, 50 μM of hemin, 5 mM H2O2, and 250 μM NADH, (**C**) The PGM signal readout when detecting different components of G4/hemin-based NADH oxidation reaction reproduced with permission from Ref. [2], copyright 2025 Elsevier B.V.).

The authors [2] have developed a universal biosensor for the detection of DNA/RNA viruses and protein particles based on DNAzymes. DNAzymes are single-stranded DNA molecules with catalytic activity. The authors found that a specific DNAzyme with a G-quadruplex (G4) structure exhibits peroxidase-like activity in the presence of hemin, catalyzing the oxidation of NADH to NAD^+^ and causing a decrease in the PGM signal (Figure 15). By integrating this strategy with CRISPR/Cas12a-mediated target recognition, the authors have successfully created a simple and versatile platform (CaG-PGM) for biosensory analysis of the monkeypox virus, model RNAs, and SARS-CoV-2 RNAs. The researchers also demonstrate that the platform can be used to detect non-nucleic acids, including proteins and small molecules.

While many of the cited studies validated their PGM-based assays using real environmental or food matrices (e.g., milk, river water, fruit extracts), a critical limitation remains the insufficient characterization of how matrix complexity influences biosensor performance. Natural water samples often contain high levels of humic acids and other organic matter that can adsorb onto enzyme surfaces or nanozymes, altering their catalytic efficiency or blocking active sites—particularly for invertase, glucose oxidase (GOx), and metal–organic framework (MOF)-based nanozymes. Similarly, agricultural products such as fruits, vegetables, and dairy inherently contain endogenous glucose, ascorbic acid, and polyphenolic antioxidants that can generate significant background signals by either directly interacting with PGM test-strip mediators or inhibiting enzyme activity. Although magnetic separation is widely used to mitigate such interference, its practicality in true point-of-care settings is questionable: it often requires precise washing steps, stable magnetic racks, and frequently complementary centrifugation to remove viscous or particulate-rich supernatants, which are rarely available in field conditions. A cross-platform review of these issues reveals a recurring trade-off: while sophisticated signal amplification and separation strategies enhance analytical sensitivity in controlled lab environments, they simultaneously undermine robustness and simplicity in real-world deployment. Future designs must prioritize matrix-resilient biorecognition elements (e.g., engineered enzymes or shielded nanozymes) and integrate passive sample cleanup (e.g., filtration membranes or hydrogel barriers) to enable reliable PGM operation without ancillary equipment.

**Table 2:** PGM-based determination of the biological ecotoxicants in the environment.

Sensor Type/Enzyme	Conjugate Type	Format of Assay	Stages of Analysis	LDR/LOD	Required Equipment	Temperature Sensitivity	POC Readiness	Sensor Characteristics		Performance Metrices	Target Object	Ref.
								Adv.	Disadv.			
Bacterial cells and DNA												
Enzymatic/invertase	Aptamer-conjugated invertase	aptamer-based assay	- Synthesis of aptamer-conjugate; - capture bacteria; - sucrose is hydrolyzed by invertase	1.2 × 10^5^ –1.2 × 10^8^ CFU·mL^−1^/ 1.0 × 10^5^ CFU·mL^−1^	U, V	Ambient 37 °C (catalysis)	No	C, D	L, M, P, R	(2) 7 interferents tested	*Staphylococcus aureus* model solution	[89]
Enzymatic/invertase	Magnetic nanoparticle-probe P aptamer	hybridization chain reactions (HCR)	- Aptamer is immobilized on a magnetic bead by hybridization with a P capture probe; - amplification of HCR; - sucrose is hydrolyzed by invertase	3–3 × 10^3^ CFU·mL^−1/^ 2 CFU·mL^−1^	W, X	Controlled 37 °C (binding), 50 °C (catalysis)	No	A, E	M, N, O, Q, R	(2) 3 interferents tested	*Staphylococcus aureus* peach juice, milk, and water samples	[90]
Enzymatic/invertase	Magnetic nanoparticle-probe H	HCR	- obtaining the MBs-H probe complex; - separating the MB-ssDNA complex; - addition of invertase-labeled H1 and H2;- sucrose is hydrolyzed by invertase	–/ 2 CFU·mL^−1^	W, X	Controlled 75 °C (binding), 50 °C (catalysis)	No	A, E	N, O, P, Q, R	(2) 3 interferents tested	*Methicillin-resistant Staphylococcus aureus* *(MRSA)*	[91]
Non–Enzymatic	scaffold containing repeated aptamer sequences and DNA sequence with sucrase on its terminal	Transcription aptamer sequences in cell recognition	- Aggregation of target bacteria based on aptamer recognition; - Isolation of *Pseudomonas aeruginosa* using low-speed centrifugation (5000 rpm); - signal amplification; - catalyze sucrose by supernatant	–/ 36 CFU·mL^−1^	V	Ambient 30 °C	No	A, B, E, F	N, O, R	(2) 3 interferents tested	*Pseudomonas aeruginosa*	[94]
Enzymatic/invertase	invertase-ssDNA-MBs	CRISPR/Cas12a-RAA with lateral flow strips (LFS)	- extracted DNA genome from Salmonella and amplified by RAA; - System launch CRISPR/Cas12; - used LFS and PGM with hydrolyzed sucrose by invertase	2.0 × 10^7^ –2.0 × 10^1^ CFU·mL^−1^/ 2.0 × 10^1^ CFU·mL^−1^	W, X	Controlled 37 °C (Cas12a reaction), 52 °C (invertase-sucrose)	No	A, B, E	N, O, Q, R	(2) 4 interferents tested	*Salmonella typhimurium* in products	[95]
Non–Enzymatic/CeO_2_ NPs	–	PCR	- Obtaining DNA amplicons by PCR; - DNA amplicons bind to CeO_2_ NPs via electrostatic interaction; - NPs-catalyzed glucose oxidation reaction	0–10^4^ copies·μL^−1^/ 10 copies·μL^−1^	Z	Ambient 20 °C (invertase-sucrose)	No	A, C, E, F, G	M, O	(2) 1 interferent tested	DNA amplicon *Escherichia coli*	[96]
Enzymatic/invertase	Magnetic nanoparticle clusters (MNCs) with IgG	sandwich immune complex	- synthesis of monoclonal antibody-functionalized magnetic nanoparticle clusters (MNCs); - capture Salmonella bacteria; - magnetic separation and washing; - conjugated to poly- - clonal antibody-functionalized invertase; - sucrose is hydrolyzed by invertase	Up to 100 CFU·mL^−1^ 10 CFU·mL^−1^	W, X	Controlled 40 °C	No	A, E, G	M, N, O, Q	(2) 3 interferents tested	*Salmonella typhimurium* in milk	[97]
Enzymatic/invertase	Invertase-MNPs-IgG	sandwich immune complex	- synthesis of conjugates Invertase-Fe_3_O_4_-IgG; - preparing an absorbent layer with sucrose; - formation of an immune complex; - sucrose is hydrolyzed by invertase	2.08 × 10^5^– 2.08 × 10^8^ CFU·mL^−1^/ 1.04 × 10^4^ CFU·mL^−1^	W, X	Controlled 50 °C	No	A, B, C, E, G	O, Q	(2) 9 interferents tested; (3) 5.7–9.5%	*E. coli* O157:H7	[98]
Enzymatic/glucose oxidase	Ampicillin (Amp) functionalized MNPs (Amp-MNPs); GOD@ZIF-8@Ab	sandwich immune complex	- Preparation of the *L. monocytogenes* complex with Amp-MNPs (*L. monocytogenes*@Amp-MNPs) by magnetic separation; - interaction with GOD@ZIF-8@Ab; - catalyze glucose decomposition	10^1^–10^6^ CFU·mL^−1^/ 10^1^ CFU·mL^−1^	V, W, X	Controlled 37 °C	No	A, B, E	N, O, Q, R	(2) 5 interferents tested	*Listeria monocytogenes* in environmental and food samples	[99]
Enzymatic/myokinase, hexokinase, puruvate kinase	–	FACER platform	cascade of enzymatic reactions	0–100 pM/ 4.8 pM	X	Controlled 45 °C	No	A, C, D, E, F, G	M	(2) 10 interferents tested; (3) RSD 2.3%; (5) 99.9–102.4%	*Chlamydia trachomatis* in human serum and urine samples	[119]
**Viruses**												
Non–Enzymatic/NADH/NAD^+^	G4/hemin DNAzyme-based complex	CRISPR/Cas12a; LAMP (RT-LAMP)	- oxidation NADH by complex G4/hemin DNAzyme-based complex; - signal registration by PGM	0.0001–0.1 U·mL^−1^/ 6.5 × 10^−5^ U·mL^−1^	None	Ambient 25 °C	Yes	A, B, C, E, F, G	O	(2) 4 interferents tested	Model RNA; SARS-CoV-2; Monkeypox virus DNA in swab samples	[2]
Enzymatic/invertase	invertase-encapsulated DNA hydrogel	CRISPR/Cas12a -derived biosensing plat- form	- preparation of the invertase-encapsulated DNA hydrogel; - pre-amplification via RCA process; - the - activation of Cas12a; - dissociation of DNA hydrogels; - sucrose is hydrolyzed by invertase	1.0 × 10^−4^ to 1.0 μg·L^−1^ 3.2 × 10^−5^ mg·L^−1^	W, X	Controlled 37 °C	No	A, B, E, F, G	N, O	(1) 14 d.; (2) N-gene agains random sequences and mismatches tested; (3) RSD 3.7%	SARS-CoV-2 N-gene in saliva, human serum	[67]
Enzymatic/glucoamylase	Streptavidin-coated Fe_3_O_4_ NPs with biotinylated capture antibody (Ab_1_); Al_2_O_3_ NPs with antibody (Ab_2_) and glucoamylase.	sandwich immune complex	- synthesis of conjugates Fe_3_O_4_ NPs and Al_2_O_3_ NPs; - formation of an immune complex; - starch was con- - verted into glucose by glucoamylase	0.2–1.76 ng·mL^−1^ (“ay”); 0.2–1.75 ng·mL^−1^ (“ad”)/ 0.3 ng·mL^−1^ (“ay”); 0.4 ng·mL^−1^ (“ad”)	V, W, X	Controlled 37 °C (immunoassay) 70 °C (enzyme conversion)	No	A, E	M, N, O, Q, R	(2) 5 interferents tested	Hepatitis B surface antigen (HBsAg)	[101]
Enzymatic/invertase	Biotinylated aptamer-oligo-invertase complex with Fe_3_O_4_ NPs	aptamer-based competitive assay.	- conjugation of invertase with the antisense oligomer strand; - conjugation of aptamer/antisense-invertase complex and - Fe_3_O_4_ NPs; - magnetic separation; - sucrose is hydrolyzed by invertase.	3.5–226 pM (Protein N); 2–128 pM (Protein S)/ 1.50 pM (Protein N); 1.31 pM (Protein S)	W, X	Controlled 60 °C (invertase- sucrose hydrolysis)	No	A, B, E, G	N, O, Q	(2) 2 inteferents tested	Protein N and S SARS-CoV-2	[112]
Enzymatic/invertase	Probe1−invertase; Probe2	sandwich DNA	- conjugation of Probe1with invertase; - DNA hybridization with complex sandwich structure - (Probe1−invertase and target and Probe2); - magnetic separation; - sucrose is hydrolyzed by invertase.	0.1–20 nM/ 98 pM (5.9 × 10^7^ copy·μL^−1^	V, W, X	Controlled 37 °C (invertase- sucrose hydrolysis)	No	A, B, D, E	N, Q, R	(1) 20 d. 4 °C 96.7% retention; (2) 6 interferents tested; (5) 97.0–121.8%	Target Nucleic Acid SARS-CoV-2 in Serum and Saliva Samples	[113]
Enzymatic/invertase	DNA-invertase	Competitive DNA	- DNA-invertase conjugation; - competitive binding of the target DNA; - magnetic separation; - sucrose is hydrolyzed by invertase	100 pM–100 nM/ 100 pM	V, W, X	Controlled 37 °C	No	A, C, D, E	M, Q, R	(2) 1 interferent tested	Model DNA	[114]
Enzymatic/invertase	ssDNA-conjugated invertase on MBs	CRISPR Cas12a-derived biosensing plat- form	- pretreatment and isothermal - amplification; - CRISPR Cas12a system with invertase-modified MBs, - sucrose is hydrolyzed by invertase.	10–10^4^ copies·μL^−1^/ 10 copies·μL^−1^	W, X	Controlled 37 °C (CRISPR reaction); 42 °C (RT-RAA amplification)	No	A, B, E	N, O, Q, R	(2) 5 interferents tested	Nucleocapsid protein gene SARS-CoV-2	[116]
Enzymatic/myokinase, hexokinase, puruvate kinase	–	CRISPR Cas13a-derived biosensing plat- form	cascade of enzymatic reactions	0–5 nM/ 27 pM	X	Controlled 37 °C (CRISPR reaction); 35 °C (CER involving myokinase)	No	A, E, F, G	M, N, O	(2) 2 interferents tested; (3) CV < 8.69%; (5) 97.41–104.09%	RNA SARS-CoV-2 human serum, plasma saliva	[117]
Enzymatic/invertase	invertase-RNA-biotin or invertase-DNA-biotin	CRISPR LAMP-Cas12a/ILR	- sample lysis; - preamplification; - cas12a/ILR reaction; - sucrose is hydrolyzed by invertase.	–/ 2.5 pM	W, X	Controlled 37 °C (CRISPR reaction); 60 °C (LAMP amplification)	No	A, B, E	N, O, Q, R	(1) 36 d. 30 °C; (2) 2 interferents each; (3) 11.8–14.9%	DNA of cervical cells	[118]

**Required Equipment U**—pH meter, **V**—Centrifuge, **W**—Magnetic rack, **X**—Thermostat, **Y**—Potentiostat, **Z**—PCR cycler **Sensor Characteristics Advantages:** A—Low LOD/High number of CFUs, B—Wide LDR, C—Easy conjugate preparation steps, D—Low cost of conjugate preparation, E—Validated matrix effects, F—Magnetic separation is not required, G—Centrifugation step is not required **Disadvantages:** L—High LOD/Low number of CFUs, M—Narrow LDR, N—Complex conjugate preparation steps, O—High cost of conjugate preparation, P—Unvalidated matrix effects, Q—Magnetic separation is required, R—Centrifugation step is required **Performance Metrices:** (1)—Stability-d.: number of days, (2)—Specificity/Selectivity, (3)—Reproducibility (reported RSD), (4)—Repeatability (reported RSD), (5)—Recoveries.

**Figure 16:**
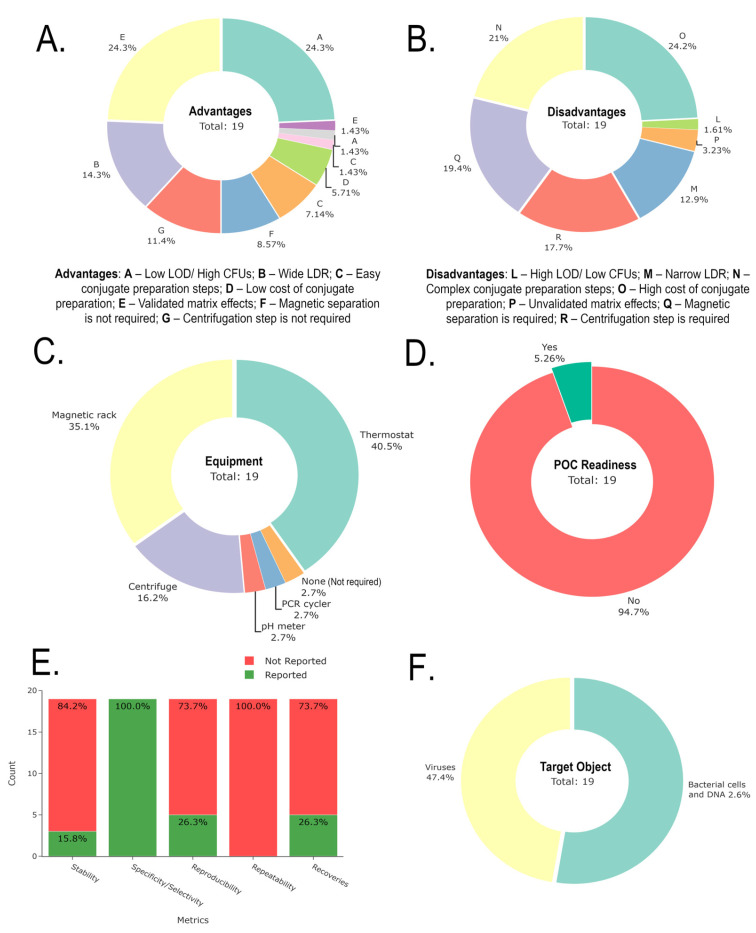
Percentage summary plots for the reviewed PGM-based sensors for the determination of biological ecotoxicants presented in Table 2. (**A**) individual advantage codes, (**B**) individual disadvantage codes, (**C**) requirement of additional equipment, (**D**) POC readiness, (**E**) performance metrices reported by the studies, and (**F**) type of biological ecotoxicant determined by the studies.

Figure 16 illustrates a summary of the advantages, disadvantages, required equipment, POC readiness, and performance metrics for all PGM-based biosensors used for biological ecotoxicants. Only 2.70% of PGM-based biosensors do not require specialized equipment. Furthermore, only 5.26% have achieved full POC readiness, representing a current limitation that needs improvement for future applications. The individual codes represent the relative percentage of each coded advantage or disadvantage, helping to identify the most prevalent features across the studies. In other words, individual percentages (feature prevalence) are the number of times individual code appears divided by the total code instance across all studies.

### 3.3. Comparative Assessment of Signal Amplification Strategies for PGM Base Nucleic Acid Detection

The integration of nucleic acid amplification and signal transduction modules has dramatically expanded the sensitivity of PGM-based biosensors for pathogenic DNA/RNA. Despite their diversity, the CRISPR-Cas (Cas12a/Cas13a), rolling circle amplification (RCA), hybridization chain reaction (HCR), and terminal deoxynucleotidyl transferase (TdT)-based strategies reviewed here share several common features when adapted to PGM: (i) they all ultimately convert target recognition into glucose signal generation or suppression Via invertase release or cascade enzymatic reactions; (ii) they rely on solid-phase separation (typically magnetic beads) to isolate the signal-generating complex from the sample matrix, minimizing background from endogenous glucose; and (iii) they require multi-step incubations that are highly sensitive to temperature and buffer composition, limiting robustness under field conditions.

CRISPR-Cas systems (Cas12a [63,95,116,118]; Cas13a [117]) offer ultra-high sensitivity and programmability but depend on expensive guide RNA synthesis, isothermal pre-amplification (e.g., RAA), and precise incubation making them better suited for near-POC or mobile lab use than true field deployment. RCA [67,94] HCR [90,91] provide strong signal amplification without enzymatic pre-amplification but still require 60–120 min of controlled hybridization, multiple washing steps, and magnetic separation—limiting speed and simplicity. TdT-mediated extension [59] achieves exceptional sensitivity through polyA tailing but suffers from the longest protocol (>3 h) and complex probe design. In contrast, washing-free strategies like FACER [119] which uses flap endonuclease (FEN1) to trigger a glucose-consuming enzymatic cascade in a single tube eliminate magnetic separation, operate at room temperature, and complete in under 30 min, representing the most promising path toward true POC compatibility (Table 3). Future efforts should prioritize such separation-free, equipment-free, and rapid designs to bridge the gap between lab-grade sensitivity and field-grade practicality.

**Table 3:** Comparative overview of nucleic acid signal amplification strategies in PGM-based biosensors for biological ecotoxicants.

Strategy	Core Mechanism	Glucose Signal	Total Assay Time	Field Compatibility	Key Limitations	Key Equipment Required	Cost Estimate *	Ref.
Cas12a	Target-activated ssDNA cleavage → invertase release	Glucose signal increases with target	60–120 min	Moderate	Requires RAA, guide RNA, thermostating	Thermomixer (37–52 °C)	High	[63] [95] [116] [118]
Cas13a	Target RNA → trans-RNA cleavage → AMP cascade	Glucose signal decreases with target	75–130 min	Moderate	Multi-enzyme cascade, sensitive to RNases	Thermomixer (35–37 °C)	High	[117]
RCA	Circular template → concatemer → aptamer display	Glucose signal increases with target	90–180 min	Low	Long incubation, magnetic separation needed	Heat block (30–37 °C)	Medium	[67] [94]
HCR	Target-initiated DNA hairpin polymerization	Glucose signal increases with target	80–150 min	Low	Probe design complexity, washing required	Heat block (30–37 °C)	Medium	[90] [91]
TdT	Target-triggered polyA tailing → invertase capture	Glucose signal increases with target	≥190 min	Low	Very long protocol, low throughput	Thermomixer, precise timing	Medium-High	[59]
FACER (washing-free)	FEN1 cleavage → AMP → glucose-consuming cascade	Glucose signal decreases with target	20–30 min	High	Limited target scope (needs flap structure)	None (room temp)	Low	[119]

* Relative reagent cost, low (<$5/test), medium ($5–15/test), high (>$15/test), determined based on enzyme/nanomaterial pricing in cited studies.

## 4. Comparative Analysis of Key Biosensing Strategies

While the diversity of PGM-based platforms demonstrates the versatility of this repurposing strategy, a critical evaluation of their core transduction and separation components reveals significant trade-offs that dictate their suitability for real-world deployment.

### 4.1. Enzymatic Versus Nanozyme Based Glucose Transduction

Traditional biosensors rely heavily on natural enzymes—particularly invertase and glucose oxidase (GOx) to generate or consume glucose in response to the target analyte. Although highly specific and efficient under controlled conditions (e.g., pH 7.4, 37 °C), these enzymes suffer from poor thermal stability, sensitivity to inhibitors (e.g., heavy metals, phenolic compounds), and limited shelf life, especially in resource-limited settings (Table 1, columns “Disadv.”). In contrast, nanozymes, such as Co-MOFs or CeO_2_ nanoparticles offer superior robustness across a wider pH and temperature range, lower production costs, and resistance to denaturation. However, their catalytic efficiency is often lower than natural enzymes, and synthetic reproducibility remains a challenge [49]. In field contexts, nanozyme-based systems thus offer greater ruggedness at the expense of absolute sensitivity and signal linearity.

### 4.2. CRISPR-Cas Versus Aptamer Switch Signal Amplification

CRISPR-Cas systems (e.g., Cas12a, Cas13a) enable extraordinary sensitivity (down to fM or single-copy levels) by coupling target recognition with non-specific collateral cleavage, thereby amplifying the signal exponentially [63,95,116,118]. Yet, this gain comes with high operational complexity: most CRISPR-PGM assays require pre-amplification (e.g., RAA, LAMP), precise reaction buffers, and multiple incubation steps (Table 2, entries [63,95,116,118], them unsuitable for rapid on-site use without trained personnel. Conversely, aptamer-switch or competitive displacement assays (e.g., for ochratoxin A [34,71] or kanamycin [26]) are simpler, often requiring only one binding and one enzymatic step, but typically achieve only nM–pM detection limits. Thus, while CRISPR systems excel in clinical or confirmatory settings, aptamer-based platforms are better aligned with frontline screening needs.

### 4.3. Magnetic Separation Versus Separation Free Designs

Nearly 70% of the assays listed in Table 1 and Table 2 employ magnetic nanoparticle (MNP)-based separation to reduce matrix interference, a necessity when analyzing complex samples like milk, soil, or wastewater (see “Disadv.”). However, this introduces a critical dependency on external magnets and, in some cases, centrifugation or buffer washing, which compromises true point-of-care (POC) applicability. Separation-free strategies, such as direct enzyme inhibition [50,51] or FACER [119] avoid this bottleneck and are faster and more field-compatible, but are vulnerable to background signals from endogenous glucose, reducing agents, or pH variations. The ideal compromise may lie in integrating passive filtration (e.g., paper microfluidics) or encapsulation systems (e.g., hydrogels, liposomes [70,99] that provide In Situ cleanup without manual washing. Collectively, these comparisons underscore a recurring theme: the inverse relationship between analytical performance and operational simplicity. Future PGM biosensor development must prioritize modular designs that allow users to select the appropriate balance, e.g., a high-sensitivity CRISPR-MNP format for laboratory validation versus a robust, separation-free nanozyme strip for field triage.

### 4.4. Systematic Comparison of Key Performance and Practical Metrics

While this review catalogs a wide array of PGM-based biosensors for environmental toxicants, a rigorous cross-platform comparison reveals critical trade-offs between analytical performance and field deployability. In terms of sensitivity, CRISPR-Cas systems (e.g., [63,116,117,118] achieve the lowest limits of detection—often in the fM to single-copy range—followed by TdT- or RCA-based amplification strategies (pM; [59,67]. In contrast, direct enzymatic assays for pesticides [50,51] or aptamer-switch platforms for antibiotics [25] typically operate in the nM to µg·L^−1^ range, sufficient for regulatory screening but not ultratrace analysis. Regarding robustness, assays relying on natural enzymes (invertase, GOx) are highly susceptible to denaturation by pH shifts, temperature fluctuations, or environmental inhibitors (e.g., phenolics, heavy metals), whereas nanozyme [49] and DNAzyme [2,72] systems demonstrate superior stability across variable field conditions and eliminate the need for cold-chain storage. Reaction complexity further distinguishes lab-bound from field-ready designs: high-sensitivity assays often require 4–6 steps, including magnetic separation, multi-temperature incubations, and long total times (60–230 min), while the most practical formats—such as cholinesterase inhibition for pesticides [50], alkaline phosphatase-based detection [51], or the wash-free FACER platform [119] involve ≤2 steps, no separation, and completion within 20–30 min. Finally, equipment requirements highlight a stark divide: over 80% of assays necessitate ancillary tools—thermostats (63%), magnetic racks (70%), or centrifuges (30%)—rendering them incompatible with true point-of-care use. Only three studies [2,50,51] require no additional equipment, functioning entirely with a standard PGM and test strips under ambient conditions. Collectively, this systematic analysis confirms that ultra-high sensitivity is rarely compatible with field practicality, and future innovation must prioritize simplicity, robustness, and equipment-free operation to enable real-world environmental monitoring.

## 5. Conclusions

The personal glucose meter is a powerful, independent, inexpensive, and portable platform that is revolutionizing the detection of ecotoxicants directly in environmental samples. This review has detailed the significant expansion of PGM’s capabilities beyond glucose, enabling the on-site quantification of a diverse range of non-glucose analytes and eliminating the reliance on centralized laboratories with their expensive instrumentation and specialized staff. A key to this versatility lies in the strategic use of highly specific bioreceptors, with aptamers, DNA, and RNA emerging as the dominant choices due to their stability and ease of modification, while antibodies are used less frequently. To ensure robustness in complex samples, a prevalent design involves conjugating these bioreceptors with magnetic nanoparticles (MNPs), allowing for efficient magnetic separation that effectively minimizes matrix interference and purifies the analytical signal.

The field has moved beyond simple invertase-based glucose generation, evolving towards more sophisticated detection strategies. These include the use of NAD-dependent enzymes to produce NADH, which directly influences the PGM’s electrochemical mediator system, and the innovative application of nanozymes—stable, cost-effective nanomaterial-based catalysts that mimic enzyme activity. For achieving ultra-high sensitivity, particularly for biological targets, researchers have successfully integrated PGM with powerful nucleic acid amplification techniques and the CRISPR-Cas12a system. This synergy allows for the detection of targets as diverse as bacteria, viruses, and heavy metal ions at remarkably low concentrations. The use of DNAzymes (catalytic DNA) for detecting viral particles represents another innovative frontier.

However, a significant challenge persists: the trade-off between high sensitivity and practical field deployment. Many of the most sensitive assays necessitate complex, multi-stage protocols involving cumbersome DNA-enzyme conjugation, thermostating, and centrifugation, which contradict the PGM’s inherent advantage of simplicity. Furthermore, a critical and often overlooked aspect is the inadequate evaluation of potential interferents, such as endogenous glucose or other sample matrix components, in control experiments, which can compromise the accuracy and reliability of results.

To qualify as a true point-of-care (POC) tool, a PGM-based biosensor must satisfy four essential operational criteria: (i) no requirement for thermostatic control assays to function reliably at ambient temperatures without precise heating or cooling; (ii) no reliance on magnetic separation or centrifugation, as these steps demand external equipment and trained handling, undermining simplicity; (iii) completion within 30 min from sample introduction to signal readout, aligning with the rapid testing expectations of field users; and (iv) robustness in complex environmental matrices, including high ionic strength, natural organic matter, and critically endogenous glucose or reducing agents that are prevalent in food, soil, and wastewater samples.

The majority of studies, summarized in Table 1 and Table 2, fail to meet one or more of these conditions. Most high-sensitivity assays depend on thermostating (e.g., 37–55 °C for enzyme activity), multi-step magnetic washing and incubation times exceeding 60 min. Moreover, few validate performance in the presence of background glucose, a fundamental flaw when repurposing a glucose meter for non-glucose targets. Establishing these criteria provides a clear benchmark for future development: POC suitability should be judged not by sensitivity alone, but by adherence to these practical constraints.

Therefore, the paramount open question remains the strengthening and simplification of PGM-based sensor systems. Future efforts must focus on streamlining assay protocols by eliminating time-consuming steps and the need for ancillary equipment. Achieving this will be crucial for developing robust, true point-of-care monitoring tools that can simplify environmental surveillance, reduce the burden on accredited laboratories, and ultimately lower the overall cost of analysis, making widespread environmental protection monitoring a more attainable goal.

Looking ahead, the evolution of PGM-based biosensors will likely be shaped by three converging technological trends. First, enzyme-free catalytic systems, particularly DNAzymes and nanozymes offer a path toward enhanced robustness, extended shelf life, and reduced production costs compared to natural enzymes like invertase or GOx. Their resistance to denaturation under variable pH, temperature, and ionic strength makes them ideal for deployment in resource-limited or extreme environments. Second, the field is increasingly shifting toward one-pot, separation-free assay formats that eliminate magnetic separation, centrifugation, and multi-step washing. Strategies such as FACER, competitive displacement in homogeneous solution, or hydrogel-encapsulated signal generation exemplify this direction, prioritizing operational simplicity without sacrificing sensitivity. Third, integration of PGM platforms with mobile-phone interfaces and AI-assisted signal processing is poised to transform qualitative reading into an intelligent diagnostic tool. Smartphone cameras can capture test-strip color changes or meter displays, while machine learning algorithms can correct for matrix effects, ambient temperature drift, or user variability, turning a simple glucose meter into a connected, context-aware biosensor node. Together, these advances point toward a future where PGM-based systems are not only portable and low-cost but also autonomous, self-calibrating, and networked for real-time environmental surveillance.

Beyond assay chemistry, practical deployment will require innovations at the engineering and system integration level. The customized PGM test strips engineered with alternative mediators (e.g., osmium complexes), broader linear ranges, and built-in filters for endogenous glucose or ascorbate could dramatically improve reliability in complex environmental matrices. The integration of on-chip microfluidics offers a powerful route to automate multi-step workflows: laminar flow channels can enable metered reagent delivery, passive mixing, magnetic-bead trapping, and washing, all within a disposable, credit-card-sized device compatible with handheld meters. Future platforms should aim for modular, all-in-one cartridges that combine sample inlet, cell lysis (for pathogens), filtration, enzymatic reaction, and glucose readout in a single unit, akin to commercial pregnancy or lateral flow tests, thereby eliminating pipetting, centrifugation, and external magnets. Such engineering advances, combined with robust biorecognition elements (e.g., nanozymes, DNAzymes), will be essential to transform PGM biosensors from laboratory curiosities into deployable tools for farmers, water inspectors, and field epidemiologists.

## Data Availability

No new data were created or analyzed in this study. Further inquiries can be directed to the corresponding author.

## Abbreviations

The following abbreviations are used in this manuscript:

Ab	Antibody
ADP	Adenosine Diphosphate
ALP	Alkaline Phosphatase
AMP	Adenosine Monophosphate
AMP-MNPs	Ampicillin-functionalized Magnetic Nanoparticles
APTES	(3-Aminopropyl)triethoxysilane
AuNPs	Gold Nanoparticles
BSA	Bovine Serum Albumin
Cas12a	CRISPR-associated protein 12a
Cas13a	CRISPR-associated protein 13a
cDNA	Complementary DNA
CeO_2_	Cerium Oxide
CFU	Colony Forming Units
COVID-19	Coronavirus Disease 2019
CRISPR	Clustered Regularly Interspaced Short Palindromic Repeats
crRNA	CRISPR RNA
Cu-Enz	Cu^2+^-dependent DNAzyme
Cu-sub	Cu^2+^-dependent DNAzyme-invertase conjugate
dCTP	Deoxycytidine Triphosphate
DNA	Deoxyribonucleic Acid
ELISA	Enzyme-Linked Immunosorbent Assay
EV-Au-β-CD/INT	Extracellular Vesicle-Gold-β-Cyclodextrin/Invertase
Exo III	Exonuclease III
FACER	FEN1-Assisted Cascade Enzymatic Reaction
FAD	Flavin Adenine Dinucleotide
FEN1	Flap Endonuclease 1
G4	G-quadruplex
GDH	Glucose Dehydrogenase
GOD	Glucose Oxidase
GOx	Glucose Oxidase
HBsAg	Hepatitis B Virus Surface Antigen
HBV	Hepatitis B Virus
HCR	Hybridization Chain Reaction
HIV	Human Immunodeficiency Virus
HPV	Human Papillomavirus
ICS	Immunochromatographic Sensor
IgG	Immunoglobulin G
ILR	Immobilized Invertase-Labeled Reporter
*L. monocytogenes*@Amp-MNPs	*Listeria monocytogenes* captured by Ampicillin-MNPs
LAMP	Loop-Mediated Isotheral Amplification
LB	Luria–Bertani Medium
LDR	Linear Dynamic Range
LFS	Lateral Flow Strips
LOD	Limit of Detection
MB	Magnetic Beads/Particles
MERS-CoV	Middle East Respiratory Syndrome Coronavirus
MNCs	Magnetic Nanoparticle Clusters
MNPs	Magnetic Nanoparticles
MOFs	Metal–Organic Frameworks
MP	Maltose Phosphorylase
MRL	Maximum Residual Limit
MRSA	Methicillin-Resistant *Staphylococcus aureus*
NAD+	Nicotinamide Adenine Dinucleotide
NADH	Nicotinamide Adenine Dinucleotide (Reduced)
NOR	Norfloxacin
NPs	Nanoparticles
OFL	Ofloxacin
P capture probe	Probe Capture Sequence
PBS	Phosphate-Buffered Saline
PCR	Polymerase Chain Reaction
PGM	Personal Glucose Meter
PGM-NAAP	Personal Glucose Meter-based Nucleic Acid Assay Platform
POC	Point of Care
polyA	Polyadenine
PQQ	Pyrroloquinoline Quinone
PQQ-GDH	Pyrroloquinoline Quinone-dependent Glucose Dehydrogenase
RAA	Recombinase Aided Amplification
RCA	Rolling Circle Amplification
RT-RAA	Reverse Transcription-Recombinase Polymerase Amplification
sAb	Secondary Antibody
SA	Streptavidin
SA-MBs	Streptavidin-Magnetic Beads
SARS-CoV-2	Severe Acute Respiratory Syndrome Coronavirus 2
SELEX	Systematic Evolution of Ligands by EXponential enrichment
SP	Substrate DNA/Substrate Probe
SPCE	Screen-Printed Carbon Electrode
ssDNA	Single-Stranded DNA
TdT	Terminal Deoxynucleotidyl Transferase
TSDR	Toehold-mediated Strand Displacement Reaction
WHO	World Health Organization
ZIF-8	Zeolitic Imidazolate Framework-8
